# Greenhouse gas reporting data improves understanding of regional climate impact on landfill methane production and collection

**DOI:** 10.1371/journal.pone.0246334

**Published:** 2021-02-26

**Authors:** Pradeep Jain, James Wally, Timothy G. Townsend, Max Krause, Thabet Tolaymat

**Affiliations:** 1 Innovative Waste Consulting Services, LLC, Gainesville, Florida, United States of America; 2 Department of Environmental Engineering Sciences, University of Florida, Gainesville, Florida, United States of America; 3 United States Environmental Protection Agency, Office of Research and Development, Cincinnati, Ohio, United States of America; Texas A&M University, UNITED STATES

## Abstract

A critical examination of the US Environmental Protection Agency’s (US EPA’s) Greenhouse Gas Reporting Program (GHGRP) database provided an opportunity for the largest evaluation to date of landfilled waste decomposition kinetics with respect to different US climate regimes. In this paper, 5–8 years of annual methane collection data from 114 closed landfills located in 29 states were used to estimate site-specific waste decay rates (k) and methane collection potentials (L_c_). These sites account for approximately 9% of all landfills required to report GHG emissions to the US EPA annually. The mean methane collection potential (L_c_) for the sites located in regions with less than 635 mm (25 in) annual rainfall was significantly (p<0.002) lower than the mean methane collection potential of the sites located in regions with more than 635 mm (25 in) annual precipitation (49 and 73 m^3^ methane Mg^-1^ waste, respectively). This finding suggests that a fraction of the in-place biodegradable waste may not be decomposing, potentially due to a lack of adequate moisture content of landfills located in arid regions. The results of this evaluation offer insight that challenges assumptions of the traditional landfill methane estimation approach, especially in arid climates, that all methane corresponding to the total methane generation potential of the buried solid waste will be produced. Decay rates showed a significant correlation with annual precipitation, with an average k of 0.043 year^-1^ for arid regions (< 508 mm (20 in) year^-1^), 0.074 year^-1^ for regions with 508–1,016 mm (20–40 in) annual precipitation, and 0.09 year^-1^ in wet regions (> 1,016 mm (40 in) year^-1^). The data suggest that waste is decaying faster than the model default values, which in turn suggests that a larger fraction of methane is produced during a landfill’s operating life (relative to post-closure).

## Introduction

Municipal solid waste (MSW) landfills are one of the major source categories that are required by US federal regulations to report annual greenhouse gas (GHG) emissions. In 2018, MSW landfills reported emitting 89 million metric tons of CO_2_ equivalents, accounting for 82% of the waste sector emissions and 17% of all methane emissions in the US [1,2]. The production rate estimates for landfill gas (LFG), which is primarily constituted of methane and carbon dioxide (~50/50% by volume), are important for sizing gas collection and control systems (GCCS), forecasting potential revenue from gas-to-energy projects, and developing global and regional GHG emission inventories. In order to estimate landfill methane emissions to the atmosphere, as done in the United States Environmental Protection Agency’s (US EPA’s) Greenhouse Gas Reporting Program (GHGRP), the measured methane collected is subtracted from an estimated amount of methane produced [3]. LFG production models employ first-order decay kinetics that rely on the mass of waste disposed of in the landfill and two empirically-derived characteristics of waste decomposition [4]. These two parameters are the methane generation rate constant or waste decay rate (k) (year^-1^), and the methane generation potential (L_0_), a representation of the ultimate volume of methane generated for each ton of waste disposed of (m^3^ methane Mg^-1^ waste). Research indicates that L_0_ is affected only by waste composition [5], whereas k can be affected by waste composition [6], landfill operations [7–9], and environmental conditions [10,13]. The Intergovernmental Panel on Climate Change (IPCC) specifically identifies different decay rate values based on climate (tropical or temperate) and rainfall (dry or moist) [4,11]. For state and national emission inventories, the US EPA provides different k rates based on precipitation rates (either < or > 635 mm (25 in) year^-1^) or operation as a wet landfill where the addition of liquid accelerates the decomposition of biodegradable waste [7–9,12].

In an effort to develop more reliable LFG production estimates, laboratory and field studies have focused on measuring k and L_0_ [13]. Krause et al. [5] presented a comprehensive compilation and critical review of MSW L_0_, finding values ranging from 20 to 223 m^3^ methane Mg^-1^ waste. Evaluating waste decay rates using laboratory or pilot-scale reactors can be challenging as waste biodegradation and the resulting LFG production is highly dependent upon the environment to which the waste is exposed [6]. Since simulating the conditions of a full-scale landfill in the laboratory is difficult, researchers have attempted to utilize LFG collection data measured at full-scale landfill sites to estimate k [10,14–18]. In some cases, an L_0_ value was also estimated as part of this analysis [14,18]. The Compilation of Air Pollutant Emissions Factors (AP-42) and New Source Performance Standards (NSPS) default L_0_ and k values were derived using this approach [12]. Data from past studies are summarized in S1 Table of S1 File.

Landfills generate and emit methane-containing LFG during all phases of life: active, closure, and post-closure. Closed landfills with a functioning GCCS offer the best opportunity to examine full-scale biodegradation kinetics as the landfill is capped, and GCCS efficiency is expected to be highest among all phases [19]. Given these conditions, one can assume that the LFG collection rate after closure is at the level closest to the generation rate (i.e., collection ≈ generation). Climate is well documented to have an effect on the waste decay rate [10,11]. What has not been previously discussed is how climate promotes or hinders the realization of the landfill’s ultimate methane generation potential, L_0_. In concept, L_0_ is a static value, and the experimental conditions used to achieve that value reflect an upper limit of the generation of the methane volume from waste [20]. At full-scale landfill operation, the observed methane yield (i.e., the cumulative volume of gas collected) is expected to be less than L_0_ due to inefficiencies in GCCS operation and possible inhibitory environmental conditions. In this research, the concept of a landfill methane collection potential (L_c_), a representation of the volume of methane actually collected from a landfill, is introduced.

LFG collection data for a number of landfills have become available as part of the GHGRP, which provides a unique opportunity to evaluate large sets of data from sites across the US. A critical examination of the GHGRP database allowed for the largest evaluation to date of closed landfill methane collection rates, especially with respect to different US climate regimes. The research complements recent analyses performed for 21 landfills [16,17]. The outcomes of this analysis not only add to the dataset of published k and L_0_, the use of a larger data set of closed sites also provides an opportunity to statistically compare the decay rate and L_c_ of landfills located in different climatic zones of the US.

## Materials and methods

### Data source and site selection criteria

The US federal regulations (40 CFR 98 Subpart HH) promulgated in 2009 require MSW landfill owners to report annual methane collection rates and historical waste placement amounts [21]. Landfills that accepted waste on or after January 1, 1980, and are estimated to emit more than 25,000 metric tons CO_2_ equivalent of GHGs annually report several types of site-specific data, including annual waste placement amounts, closure status, and the amount of methane collected and destructed at the site (for the landfills with an active GCCS). These data were downloaded from the GHGRP portal (https://www3.epa.gov/enviro/). The database was accessed on 03/28/2019 and contained data from over 1,300 MSW landfill sites. The following criteria were used to select the sites for this study; S1 Fig of the Supporting Information presents more details on the approach used for selecting the sites for modeling:

1. The landfill was closed before 2013 and capped with a final or an intermediate cover; this criterion was specifically used to select sites with minimal fugitive LFG emissions.
2. The landfill is equipped with an active LFG collection system covering the entire waste disposal area;
3. At least 5 years of post-closure methane collection data are available; and
4. No collected methane collection rate anomalies were observed (i.e., yearly spikes, if any, in methane collection rate are less than 50% and a general decreasing trend). S2 and S3 Figs present methane collection rate of example sites that were excluded due to collection rate anomalies.

Data from the 127 closed sites that met the criteria listed above were analyzed to estimate site-specific LFG modeling parameters using the approaches described in the next section.

### Inverse methane collection modeling

LandGEM, an LFG modeling tool developed and maintained by US EPA, codifies the first-order waste decay and LFG generation from MSW landfills as outlined in AP-42 and the NSPS regulations [22]. The model uses the following equation for estimating methane generation rate as a function of annual waste placement data, methane generation potential, and decay rate:

$$Q_{modeled,t}=kL_{o}\sum_{i=1}^{n}\sum_{j=0.1}^{1}\left(\frac{M_{i}}{10}\right)e^{-kt_{ij}}$$ (1)

where

Q_,modeled,t_ = annual methane generation rate in the year of the calculation (m^3^ methane year^-1^)

i = 1 year time increment

n = (year of the calculation) − (initial year of waste acceptance)

j = 0.1 year time increment

k = methane generation rate (year^-1^)

L_0_ = potential methane generation capacity (m^3^ methane Mg^-1^ waste)

M_i_ = mass of waste accepted in the i^th^ year (Mg)

t_ij_ = age of the j^th^ section of waste mass M_i_ accepted in the i^th^ year.

The Microsoft Excel Solver function was used to minimize the sum of squared error (SSE) between the reported annual methane collection rate and the collection rate modeled using the first-order decay model by varying decay constant and methane generation potential values; the reported site-specific annual disposal amounts were used for modeling.

Since the analysis presented in this paper is based on the methane collection rate, the value that provided the best-modeled methane flow rate is referred to herein as the methane collection potential (L_c_) rather than L_0_. A site-specific k and L_c_ estimated using the MS Excel LOGEST function was used as initial values for SOLVER (see Supplementary information). In addition, three values for L_c_ of 20, 100, and 230 m^3^ methane Mg^-1^ waste, which reflect the wide variation in the methane generation potential reported for MSW, were used as the initial L_c_ value for inverse modeling. L_c_ was constrained to less than 223 m^3^ methane Mg^-1^ waste, which represents the upper bound of methane generation potential reported by Krause et al. [5]. The 223 m^3^ methane Mg^-1^ waste value was selected from the critical review [5] based on the highest reported L_0_ of a single waste type (i.e., corrugated cardboard) in controlled laboratory analysis. Thirteen sites with the best-fit L_c_ estimate greater than 223 m^3^ methane Mg^-1^ waste were excluded from further analysis and are given in S2 Table in S1 File. The decay constant (k) values were similarly constrained to range from 0.001 to 2.2 year^-1^ based on the range of the values reported in the literature for MSW [23]. S4 Fig presents a comparison of the reported measured data and the modeled methane collection rates based on the methodology described above.

### Variations in Lc and k with weather data

The site-specific L_c_ and k estimates were analyzed with respect to annual precipitation, average annual temperature, and the number of days below freezing derived from the weather station nearest to each landfill site using 1981–2010 Climate Normals published by the US National Oceanic and Atmospheric Administration (NOAA) which provide average weather values (precipitation, temperature, and the number of days below freezing) over a thirty-year period [24]. The analysis of variance was used for statistically comparing more than two groups of L_c_ and k estimates. Assuming unequal variances, the Welch t-Test was used to statistically compare two groups of L_c_ and k estimates.

## Results and discussion

### Landfill sites

**Fig 1:**
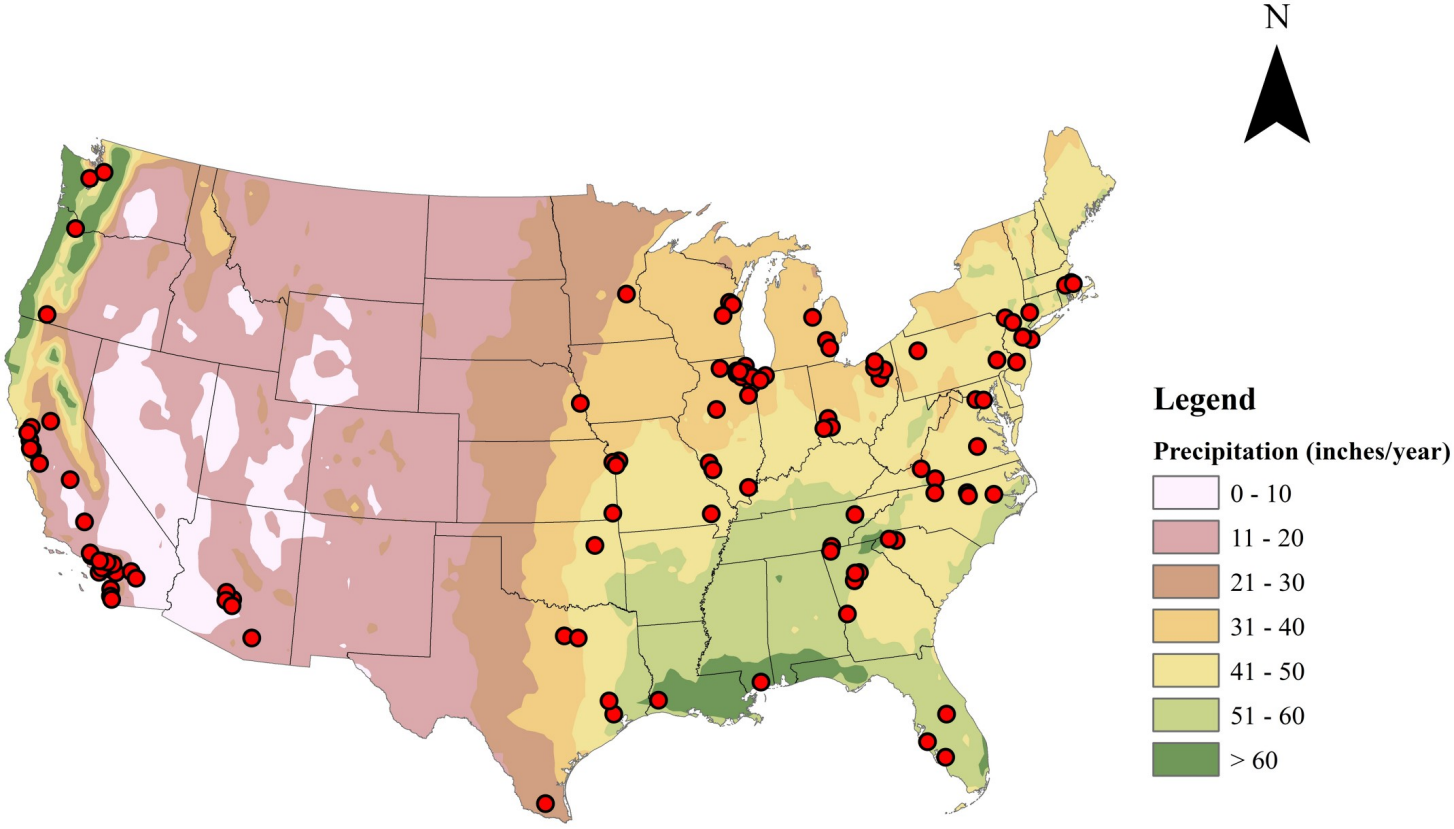
Locations of the 114 sites selected for the study overlying the distribution of annual rainfall in the US. These sites are located in 29 states of the US.

Fig 1 shows the locations of the 114 closed landfills that met the selection criteria and the distribution of annual rainfall in the US. The sites are located in 29 states and represent approximately 9% of the landfills included in the GHGRP database. Over 35% of the sites are in California (25 sites) and Illinois (16 sites). More than 55% of the sites reported annual methane collection rates for 8 years, while 30% reported for 5 years. Over 75% of the sites estimated annual waste disposal amounts (for the entire waste acceptance period or part) based on in-place waste volumes and an assumed waste density rather than direct mass measurements. Pertinent data for these sites are presented in S2 and S3 Tables of the S1 File. Over 90% of the sites started accepting waste before 1985, only two sites started after 1990, and 64% of the sites were closed after 1995.

Approximately 70% of the sites are reported to be capped with a final soil cover 0.9 m (3 ft) or thicker of clay or final cover as approved by the regulatory agency and/or geomembrane system. GHGRP assigns a collection efficiency of 95% for these final cover types though no collection efficiency assumptions were made in this analysis. The remaining 30% of sites are reported to be covered with an intermediate cover or a final cover not meeting the criteria listed above; GHG regulations assign a collection efficiency of 75% for these final cover types.

### Methane collection potential (L_c_)

The L_c_ estimates for 114 sites were found to range from approximately 19 to 221 m^3^ methane Mg^-1^ waste, with a median of 65 m^3^ Mg^-1^. As a point of comparison, the US Clean Air Act default L_0_ is 170 m^3^ Mg^-1^ (40 CFR § 60.764 (a)(1)), which is used for calculating the annual non-methane organic compound (NMOC) generation rate for determining whether a GCCS is required or not. The AP-42 default L_0_ is 100 m^3^ methane Mg^-1^ waste, which is typically used for the LFG collection system design [12]. While the median value for L_c_ was within range of the published L_0_ values [5], a large variability was observed in the estimated L_c_ values; S5 Fig presents a distribution of the estimated L_c_ values. Six of the 114 sites had an L_c_ greater than 170 m^3^ methane Mg^-1^ waste. Although some of the highest L_c_ values appear to be unrealistic for decomposing waste, these may be due to the potential uncertainty of the reported mass of in-place waste at landfill sites. In many cases (70% of the landfills), the waste in-place was estimated based on the waste volume and an assumed in-place density, which has been reported to have a wide range (240–2,350 kg m^-3^) [25]. Choosing a density on the low end of this range would result in an underestimation of waste mass, which in turn would result in an overestimation of L_c_. The GHGRP database does not include the density used for calculating mass based on volume for the study sites. Total reported in-place waste amounts for the study sites are presented in S3 Table in S1 File.

Overall, approximately 80% and 95% of the sites were found to have L_c_ less than the AP-42 recommended L_0_ values of 100 and NSPS default value of 170 m^3^ methane Mg^-1^ waste, respectively. However, it is paramount not to conflate the L_c_ derived using this methodology with the L_0_ used in the LFG models and is often based on optimized laboratory experiments [9,20,26]. As traditionally conceived, L_0_ represents the total amount of methane that will eventually be produced under conditions favorable for anaerobic decomposition and thus depends solely on the composition of the waste and not external factors in the landfill. One factor that results in a difference between L_0_ and L_c_ is that not all the LFG that is generated is captured by the GCCS as it is not 100% efficient.

While the first-order decay approach assumes that all of L_0_ is ultimately realized (i.e., all biodegradable waste components will eventually decay completely), some landfilled waste components may simply not decompose due to lack of adequate moisture content as might be expected in arid climates [10,27]. L_c_ values for some of the study sites are substantially lower than an expected L_0_, even considering GCCS inefficiencies (typically assumed to be 75–95% efficient at this stage of the landfill lifetime). Assuming insignificant variations in waste composition among the 114 sites (i.e., that all landfills have the same L_0_), low Lc values of landfills in arid climate suggest that some fractions of the waste may not be degrading in the current disposal environment.

Environmental conditions such as temperature and annual rainfall have long been assumed, and in some cases shown, to affect the waste decay rate [10]. However, the implicit assumption of using a single L_0_ across all geographic regions was that the ultimate methane production per ton of waste would be equivalent for all sites over a long time period. In other words, the rate of landfill methane generation (k) would be different, but given an equal mass of waste, the cumulative volume of LFG produced would eventually be the same, irrespective of the environmental conditions.

**Fig 2:**
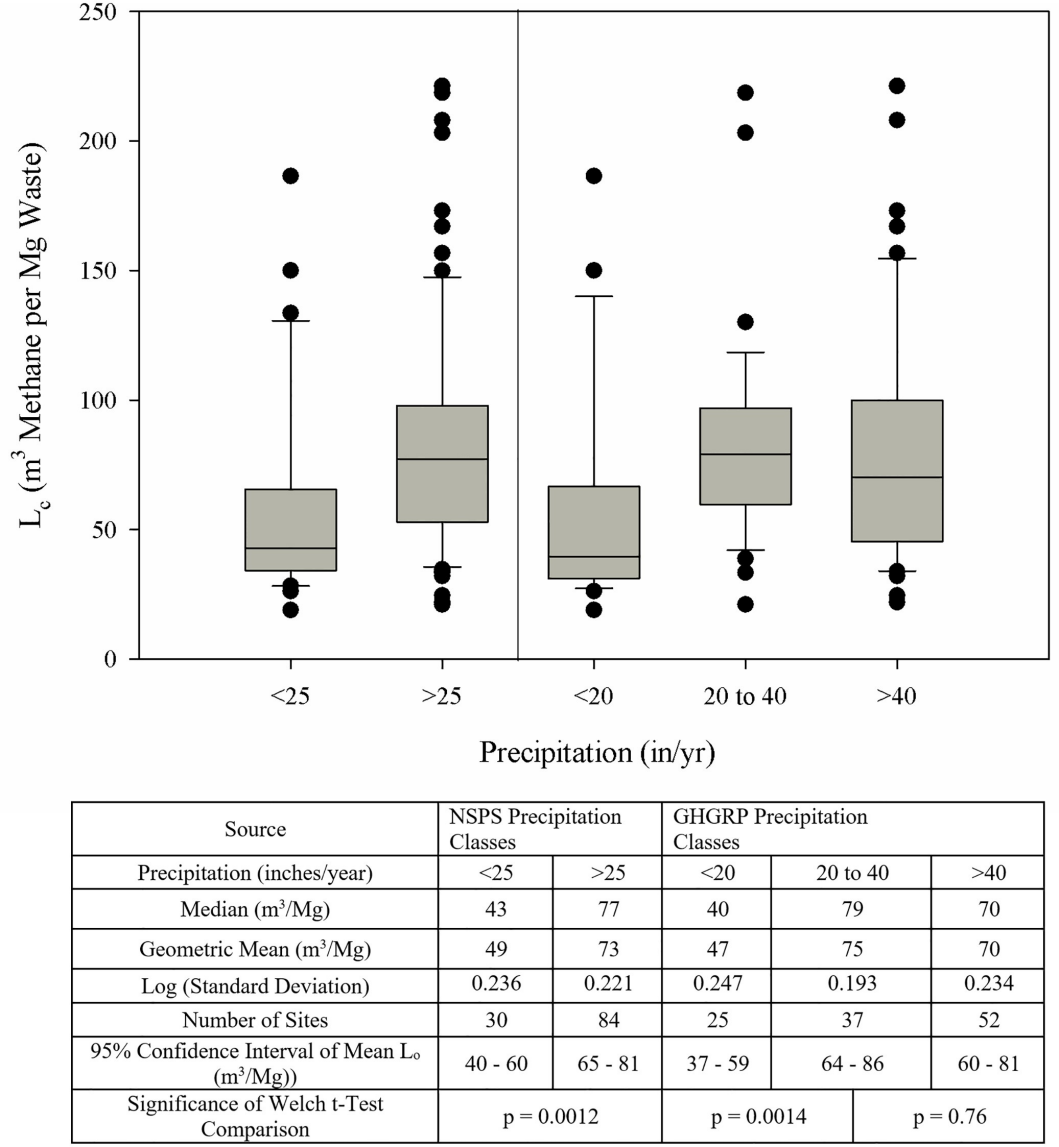
Distribution of L_c_ for the sites located in precipitation zones used by NSPS and GHGRP for MSW landfills.

Precipitation is expected to impact the moisture content of received waste as rainfall/snowmelt infiltrates the landfill. The distribution of L_c_ values as a function of precipitation zones was analyzed, as shown in Fig 2. The log-transformed L_c_ values were found to be normally distributed based on the Shapiro-Wilk normality test. The mean L_c_ for the landfills located in the arid climate (L_c_ = 49 m^3^ methane Mg^-1^ waste for areas <635 mm (25 in) annual precipitation) was significantly (p<0.002) lower than the mean L_c_ of landfills in areas with precipitation rate greater than 635 mm (25 in) (L_c_ = 73 m^3^ methane Mg^-1^ waste). Similarly, the mean L_c_ for landfills in areas with less than 508 mm (20 in) annual precipitation was significantly (p<0.002) lower than the mean L_c_ for landfills located in 508–1,016 mm (20–40 in) annual precipitation areas (L_c_ = 47 and 75 m^3^ methane Mg^-1^ waste, respectively). However, no significant difference in mean L_c_ was found between landfills in areas with 508–1,016 mm (20–40 in) and greater than 1,016 mm (40 in) annual precipitation (L_c_ = 75 and 70 m^3^ methane Mg^-1^ waste, respectively). Assuming that the composition of waste deposited in landfills in different regions of the country is similar, the analysis suggests that the landfills in arid climates appear to contain biodegradable carbon that is not degrading. This fraction could decay under more favorable conditions. No clear trends between L_c_ and the ambient temperature and days below freezing were observed for the sites in a similar annual precipitation zone (see supporting information S9–S11 Figs).

### Estimated decay rate (k)

The estimated decay rates of 114 sites ranged from 0.004 to 0.226 year^-1^, with a median of 0.068 year^-1^. S6 Fig presents the distribution of k values for 114 sites. The estimated k values of all but one of the 114 sites (which resulted in k of 0.004 year^-1^) were in the range of k reported in the literature. The higher end of the range corresponds to values typically reported for wet landfills, such as those discussed in Barlaz et al. [7] and Faour et al. [23], while the lower end correlates with values reported for arid landfills [10]. Approximately 90% of the sites were estimated to have a k within the 0.02–0.17 year^-1^ range. Only 7% of the sites resulted in an estimated k of less than 0.02 year^-1^. Approximately 70% and 83% of the sites had k values greater than 0.05 year^-1^ and 0.04 year^-1^ (the defaults for regions with <635 mm (25 in) annual precipitation). Unlike L_c_, k values estimated from the measured methane collection rate after closure are not dependent on the waste mass or collection efficiency (assuming that efficiency does not vary over the duration of measured methane collection rates used in the inverse modeling, which is a reasonable assumption for closed landfills). The k estimates based on the measured methane collection rates, therefore, are more robust than L_c_ estimates.

**Fig 3:**
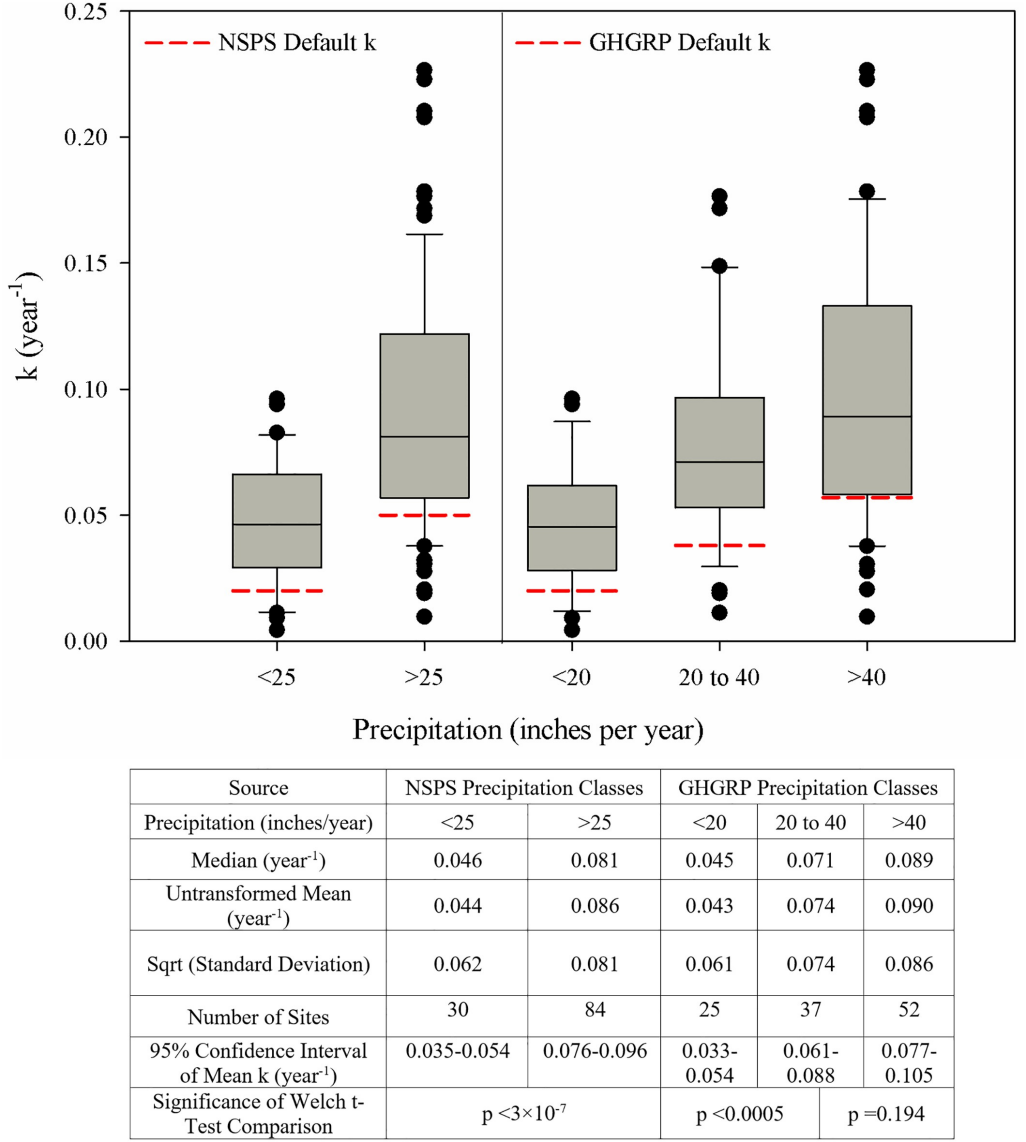
Distribution of k for the sites located in precipitation zones used by NSPS and GHGRP for MSW landfills.

An analysis of variances was conducted to assess the variability of k with weather data. Fig 3 presents the distribution of decay rates for the precipitation classes used by NSPS and GHG regulations, along with the k values specified by these regulations. NSPS specifies the use of two k values, and the GHGRP specifies three possible k values, depending on site-specific precipitation levels. More than 85% of the sites in each precipitation region had a k greater than NSPS-specified k value for the respective precipitation zone. The square-root-transformed k values were found to be normally distributed based on the Shapiro-Wilk normality test. The average of square-root-transformed k values was found to be statistically different (p<0.0001) between these sites in these two precipitation zones.

When analyzed with respect to GHGRP, approximately 88%, 89%, and 77% of the sites within each precipitation zone had a k greater than the regulatory default of 0.02, 0.038, and 0.057 year^-1^, respectively. The average k values (square-root-transformed) of sites located in a less than 508 mm (20 in) annual precipitation zone were found to be statistically lower (p<0.001) than those in regions with annual precipitation of 508–1,016 mm (20–40 in) and more than 1,016 mm (40 in). The average k of sites in the 508–1,016 mm (20–40 in) annual precipitation zone was not statistically different than the average k of sites located in the zone with greater than 1,016 mm (40 in) annual precipitation.

The addition of liquids into the landfill mass is known to stimulate decomposition and methane production [7–9]. Eleven sites reported recirculating leachate occasionally in the past 10 years. The average k values for these sites were not statistically different than the average k values of the non-recirculating sites. The volume and exact frequency of leachate recirculation were not available for detailed evaluation.

While waste decay rate has been well documented to depend on moisture, the impact of ambient temperature and freezing conditions on waste decomposition has not been established to date, primarily due to lack of decay rate estimates from an adequate number of sites [10]. S12–S14 Figs present site locations with respect to the distribution of precipitation, days below freezing, and average annual temperature in the US. IPCC suggests k values based on both precipitation and average annual temperature of a site [11]. The sites with similar precipitation were grouped into different annual average ambient temperature groups for statistical comparison to control for the impact of precipitation while assessing the impact of temperature on k within each precipitation group. No statistically significant (p<0.05) differences in k were found among different temperature sub-groups of each precipitation group. Although the decay rate is expected to be dependent on the in-place waste temperature, the waste temperature, especially in the deeper sections of the landfill, is not influenced by the ambient temperature, as reported by Yeşiller et al. [28]. Similarly, an evaluation of k with respect to the days below freezing showed no significant differences across the study sites (see supporting information S12–S14 Figs).

### Comparison of the modeling and measured methane collection rates

**Fig 4:**
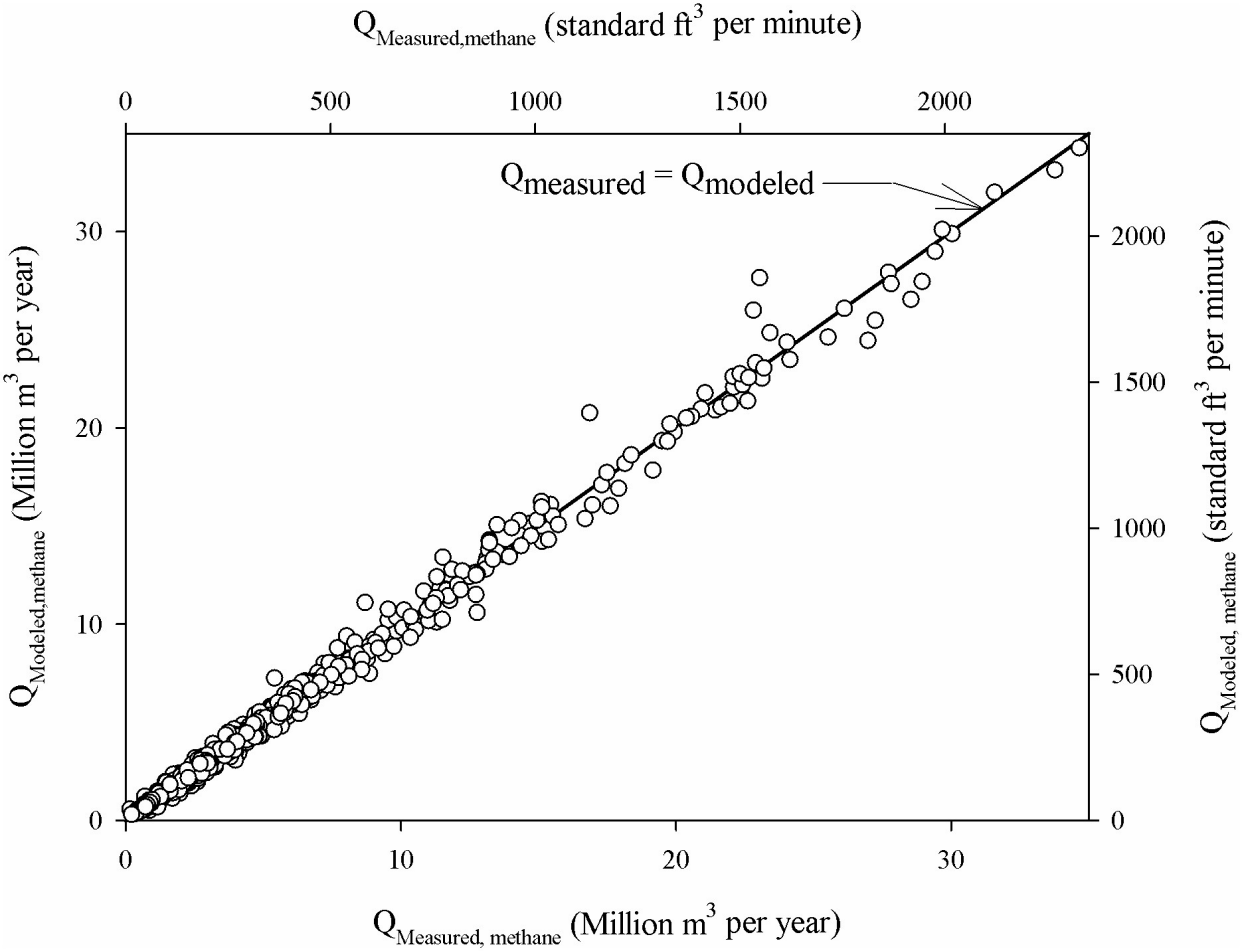
Comparison of the modeled methane collection rates corresponding to the site-specific estimates of L_c_ and k with the measured (reported) methane collection rates of 114 closed landfills. Over 94% of the modeled flow rates were within ±20% of the measured collection rates.

The site-specific estimates of L_c_ and k along with the reported annual waste mass data, were used to estimate the methane collection rate using the first-order decay model. Fig 4 presents a comparison of the modeled and measured methane collection rates for the 114 sites evaluated. An example of the modeled and measured data comparison for a site is presented in S4 Fig. The modeled methane collection rates were within ±20% of the measured rate for over 94% of the measurements available for all the sites, indicating that the estimated L_c_ and k provide a reliable estimate of these first-order decay modeling parameters for these sites.

## Implications of findings

Estimates of k based on 114 closed landfills sites confirmed the commonly held understanding that the waste decays faster in climates with greater precipitation [12]. The analysis found a median k of 0.077 year^-1^ at sites located in wetter regions of the US (>508 mm (20 in) year^-1^) and a median k of 0.045 year^-1^ for sites in drier climates with less than <508 mm (20 in) annual precipitation. The US and international landfill methane estimation methodologies assign default k values differently based on climate. A greater k value suggests more methane emission occurs earlier in the life of the landfill. Of note is that the values estimated in this evaluation are greater than model defaults. The higher decay rates suggest that more methane is produced from landfills in all climates prior to closure than currently estimated based on regulatory or AP-42 defaults, including during times when gas collection infrastructure may not yet have been installed.

The estimated higher decay rates also have implications for closed landfills. The US NSPS regulations require the construction and operation of a GCCS at landfills generating more than 50 Mg (or 34 Mg if regulated under Subpart XXX) of NMOC annually. NSPS regulations (Subparts WWW and XXX) allow termination of GCCS after the annual NMOC generation rate has dropped below 50 Mg per year (or 34 Mg per year if meeting the requirements of Subpart XXX). Given the faster decay rates suggested by this study, landfills are expected to reach the annual NMOC generation threshold more quickly than predicted based on the current defaults. The GCCS operation, therefore, can be terminated sooner than estimated based on regulatory defaults or AP-42 recommended values.

As mentioned earlier, over 1,300 MSW landfills in the US report GHG emissions annually. This domain represents both active and closed facilities. As we evaluated only closed landfills that met the study criteria, the impacts of the study findings (i.e., higher decay rates than the regulatory defaults) on the overall annual GHG emissions from MSW landfills in the US could not be readily quantified. Due to the larger number and size and lower GCCS efficiencies of active landfills than closed landfills, the estimated higher decay rates suggest that MSW landfills are emitting more methane than estimated based on GHGRP-default k values.

Given that MSW composition is not expected to vary significantly across US regions, conventional LFG modeling practice assumes that L_0_ is not a function of climate or moisture and that while landfills in wetter climates (or those operated purposefully to be wet, i.e., bioreactor landfills) would produce methane in a shorter timeframe, the cumulative methane production will be the same regardless of the climate (illustrated in S15 Fig). However, the L_c_ values from landfill sites in arid regions (<635 mm (25 in) annual precipitation) were found to be significantly lower than the L_c_ values from landfills in non-arid regions (> 635 mm (25 in) annual precipitation). It is hypothesized that the outcomes measured here result from a waste decomposition paradigm that differs from this conventional wisdom. In non-arid climates, sufficient moisture is present in the landfill for more thorough waste decomposition to occur. The L_c_ values estimated in this study for non-arid regions are similar to L_0_ values reported by other studies based on laboratory measurements from waste [20,26]. However, in drier climates, the landfills do not appear to have sufficient moisture to support biological waste decomposition, and a part of the biodegradable carbon remains undegraded.

Several implications of this alternative concept of waste decomposition in arid climate landfills emerge. On the one hand, it could be argued that some biodegradable waste deposited in the arid-region landfill is conserved within the landfill and that a lower L_0_ may be applied for collection rate and emissions estimation. Under this viewpoint, landfills in arid climates can be considered less of a methane source risk than previously thought because the moisture content of the waste is too low to support full anaerobic decomposition. An alternative viewpoint, however, is that landfills in arid climates should be considered potential long-term sources of methane. The US landfill closure rules do not, as yet, define when a landfill is considered sufficiently stable to cease post-closure care. The US regulations stipulate that once NMOC emissions decrease below an annual emission threshold, the landfill is no longer subject to the active GCCS requirements. However, there are no regulatory provisions in place to require resuming LFG collection should emissions from a site re-emerge after GCCS termination. If one were to define stable as no longer having the substantial potential to generate methane, landfills in arid climates would likely never reach stable conditions without applying operating techniques that promote complete waste decomposition/stabilization.

The results of the study suggest that landfill engineers might consider using a decay rate higher than current default values for appropriate sizing of a GCCS. The owners/operators of the landfills located in arid areas should consider operating approaches such as leachate recirculation (or addition of other liquids if needed) to promote waste decomposition to reduce potential GHG emissions after closure and post-closure care periods. The solid waste community should consider periodically updating decay rates and methane potentials based on the measured LFG collection rates and composition to accommodate the impact of changes in waste composition and landfill management practices over time on LFG generation, collection, and emission from MSW landfills.

The decay rates and methane collection potentials presented in this paper have limitations. First, the in-place waste amounts used for the inverse modeling are not based on actual measurements. Over 75% of the sites used for the analysis reported using in-place volume and assumed density to estimate the in-place mass. The density values used were not reported. The in-place waste density has been reported to range over a wide range and can have a significant impact on the accuracy of the estimated waste mass. Second, limited waste composition data were available for these sites. The bulk waste composition (including non-MSW streams such as C&D debris and other inert materials such as contaminated soil), which may vary significantly among the sites, may have a significant impact on the decay rate and the methane generation/collection potential. Finally, the estimation methodology assumed a steady LFG collection efficiency over time. Although significant temporal variation in the collection efficiency of a well-operated system is unlikely, it may vary over time. The variation in the gas collection efficiency over time may have a significant impact on the decay rate estimate. A reliable estimate of the collection efficiency is not typically available even for modern landfills because while LFG collection is measured, LFG generation is modeled. Future studies should consider using post-closure LFG collection data from modern landfills with accurate in-place waste mass and composition data to estimate the decay rates and methane generation/collection potentials. Alternatively, for all the reasons above, site-specific conditions and varying waste composition bring considerable uncertainty to these parameter values. With the advancement in remote sensing technologies, emissions from landfills may soon be able to be measured directly and reduce the need for modeling approaches for landfill GHG inventories all together [29,30].

## Supporting information

S1 Fig Flow chart depicting the site selection approach for regression and first-order decay inverse modeling. (TIF)

S2 Fig Example of a site that exhibited a generally increasing methane collection rate trend. Sites that exhibited an increasing trend for the methane collection rate were excluded from the study.
(TIF)

S3 Fig Example of a site that exhibited at least one annual increase of more than 50% in methane collection rate. These sites were excluded from the analysis. There is a 74% increase from 2011 to 2012 in methane collection rate for this site.
(TIF)

S4 Fig Example of a methane collection rate trend of a site that was selected for regression and the first-order decay inverse modeling. The plot also includes the modeled methane collection rates corresponding to the minimum SSE. The modeled data present a good approximation of the measured methane collection rates.
(TIF)

S5 Fig Distribution of the site-specific methane collection potential (L_c_) values estimated based on inverse first-order decay modeling using the reported methane collection rate and waste tonnage for the 114 sites included in the study. (TIF)

S6 Fig Distribution of the site-specific decay rate (k) values estimated based on inverse first-order decay modeling using the reported methane collection rate and waste tonnage for the 114 sites included in the study. No decay rate estimates were within 0.18 to 0.2 year^-1^.
(TIF)

S7 Fig Plot showing site-specific decay rate (k) values as a function of precipitation for the 114 study sites included in the study. Data show some degree of correlation between precipitation and k estimates.
(TIF)

S8 Fig Example residuals plot of a site. The lack of trend in the residuals and the random scatter pattern indicates a lack of bias in the modeled data. The residuals plot for each site was to confirm a lack of bias.
(TIF)

S9 Fig Location of the 114 study sites by L_c_ value compared to annual precipitation in the contiguous US. Lower precipitation regions generally had lower L_c_ estimate.
(TIF)

S10 Fig Location of the 114 study sites by L_c_ value compared to the number of days below freezing in the contiguous US. No clear trends between the days below freezing and L_c_ estimates were observed for the sites in the similar annual precipitation zones.
(TIF)

S11 Fig Location of the 114 study sites by L_c_ value compared to the site-specific average ambient temperature in the contiguous US. No clear trends between the average ambient annual temperature and L_c_ estimates were observed for the sites in the similar annual precipitation zones.
(TIF)

S12 Fig Location of the 114 study sites by k value compared to annual precipitation in the contiguous US. Lower precipitation regions generally had lower site-specific k estimate.
(TIF)

S13 Fig Location of the 114 study sites by k value compared to the number of days below freezing in the contiguous US. No clear trends between the days below freezing and k estimates were observed for the sites in the similar annual precipitation zones.
(TIF)

S14 Fig Location of the 114 study sites by k value compared to the average temperature in the contiguous US. No clear trends between the average annual temperature and k estimates were observed for the sites in the similar annual precipitation zones.
(TIF)

S15 Fig Comparison of methane generation rate trends for a bioreactor (k = 0.2 year^-1^) and conventional (k = 0.04 year^-1^) landfill with an annual disposal rate of 100,000 Mg per year, a life span of 20 years and methane generation potential (L_o_) of 100 m^3^ methane per Mg waste. The generation rate is higher for bioreactor case before closure and lower after closure than a conventional landfill.
(TIF)

S16 Fig (DOCX)

S1 File (DOCX)
