# Supplementary figures and images for: Greenhouse gas reporting data improves understanding of regional climate impact on landfill methane production and collection
Source: PLoS One. 2021 Feb 26;16(2):e0246334. doi: 10.1371/journal.pone.0246334 (PMC7909644; doi:10.1371/journal.pone.0246334)

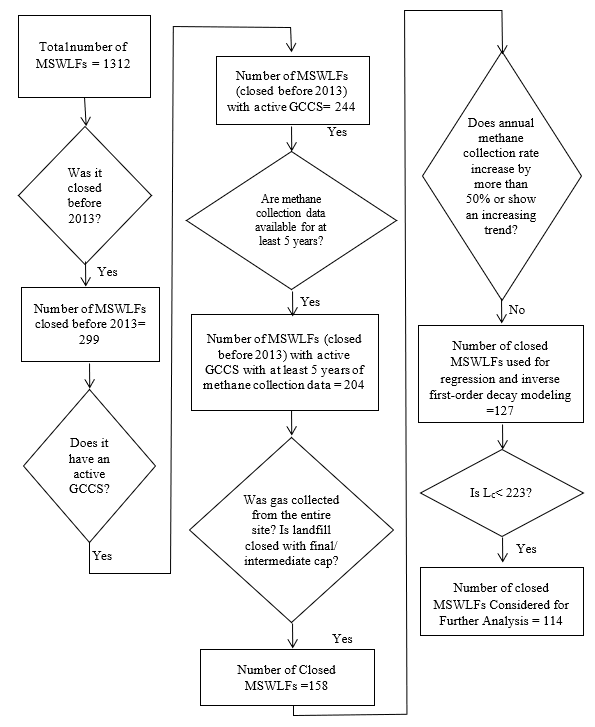

Supplement: S1 Fig — (TIF) [file pone.0246334.s001.tif]

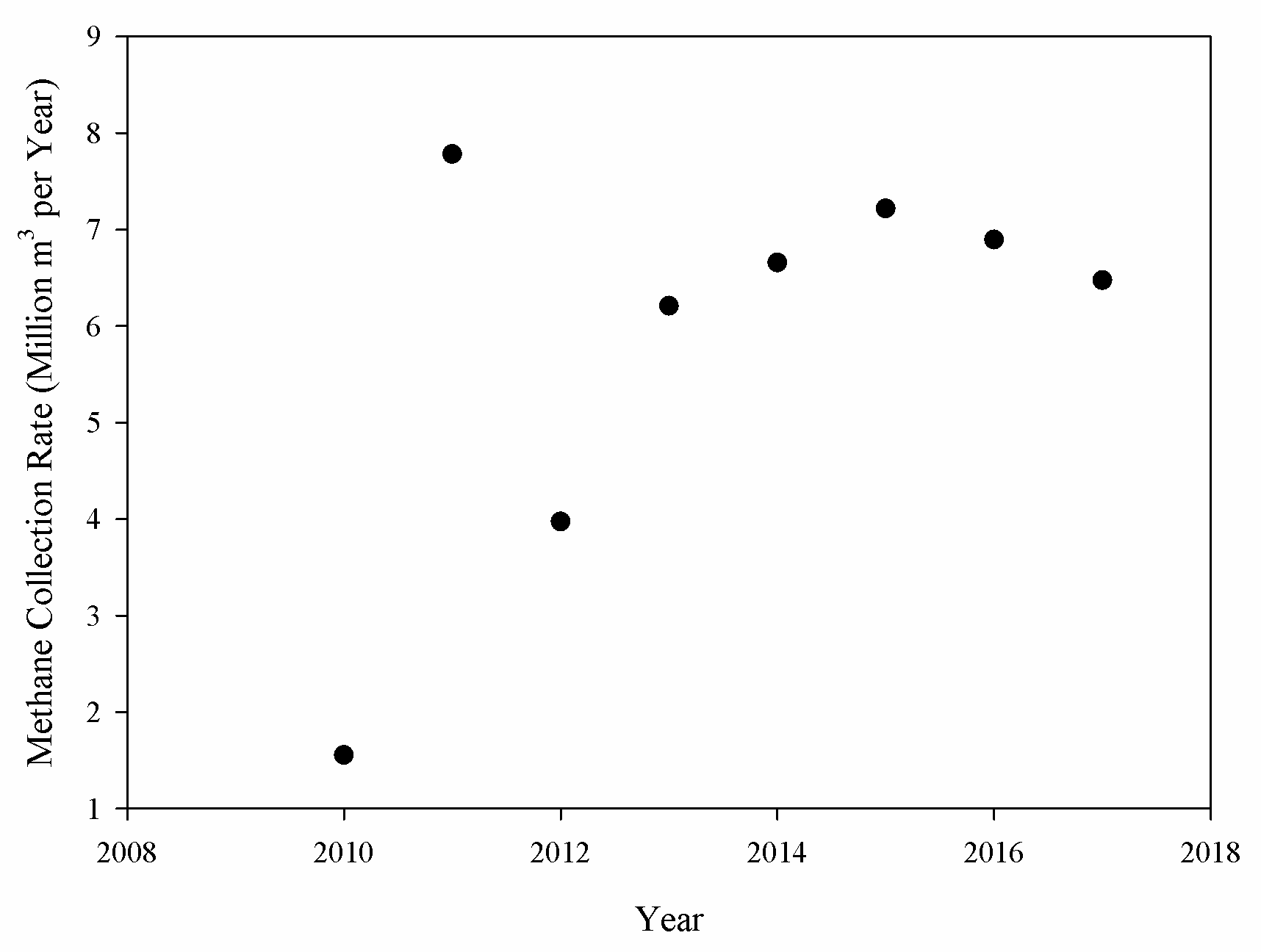

Supplement: S2 Fig — Sites that exhibited an increasing trend for the methane collection rate were excluded from the study. (TIF) [file pone.0246334.s002.TIF]

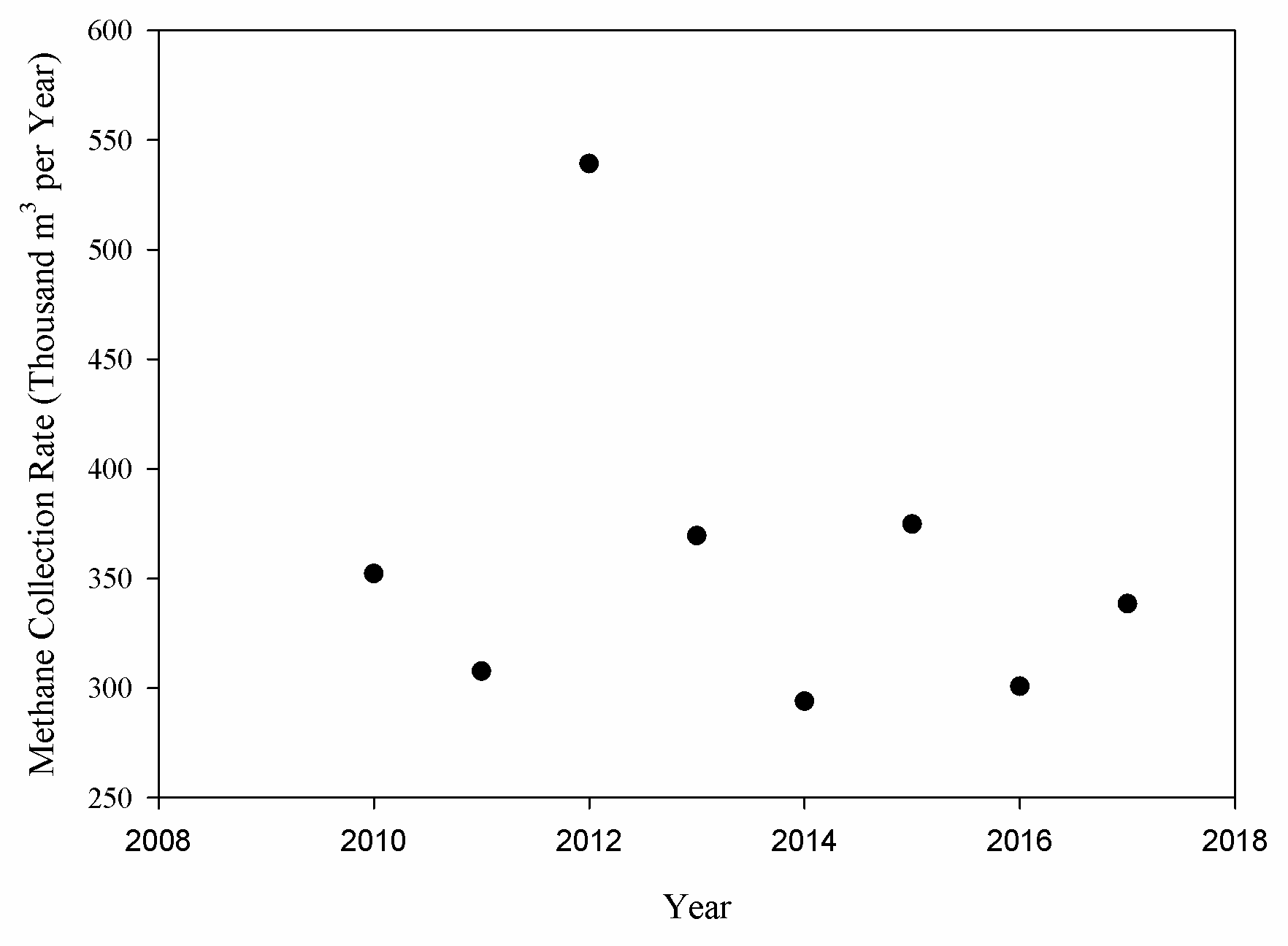

Supplement: S3 Fig — These sites were excluded from the analysis. There is a 74% increase from 2011 to 2012 in methane collection rate for this site. (TIF) [file pone.0246334.s003.TIF]

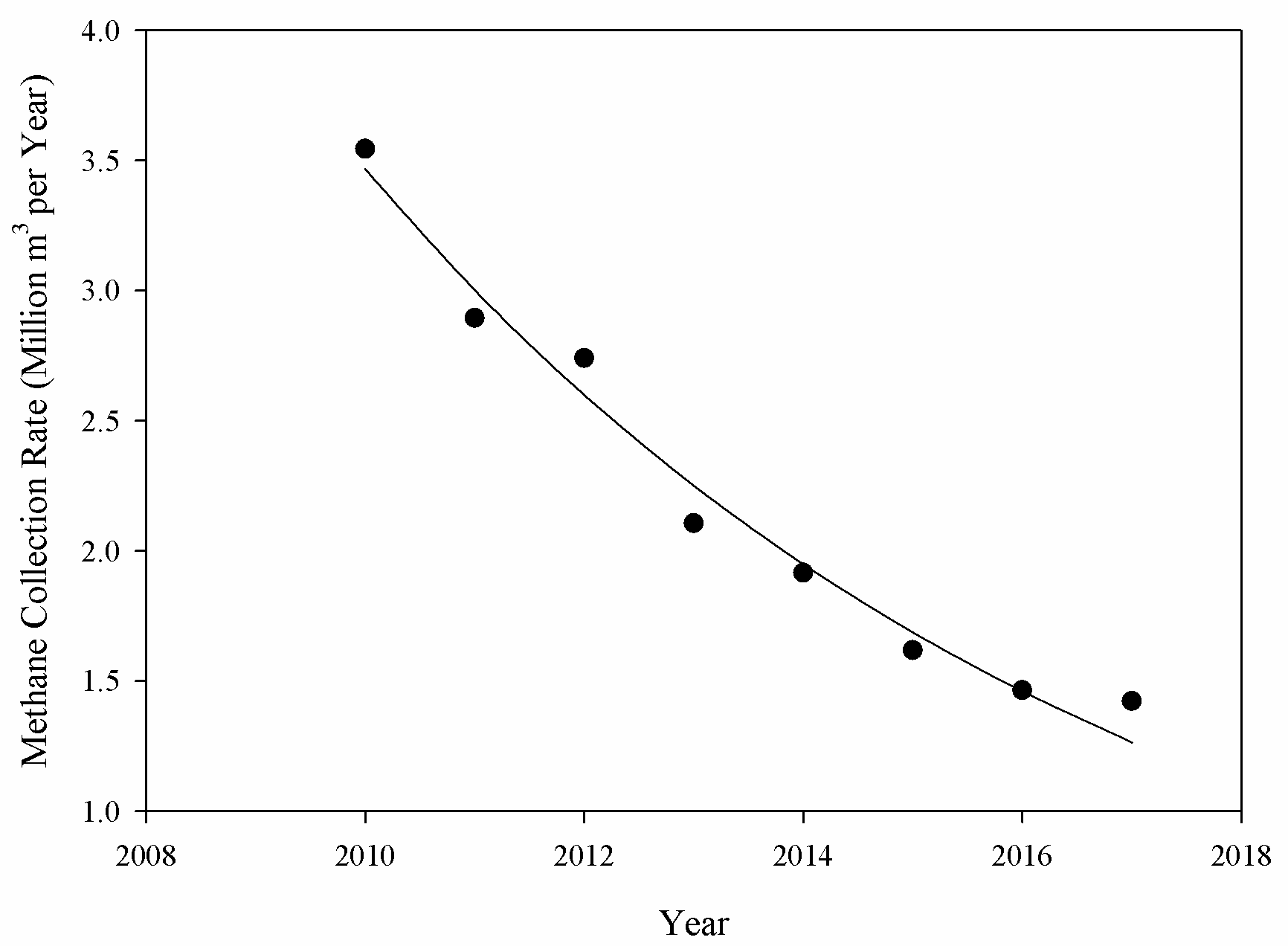

Supplement: S4 Fig — The plot also includes the modeled methane collection rates corresponding to the minimum SSE. The modeled data present a good approximation of the measured methane collection rates. (TIF) [file pone.0246334.s004.TIF]

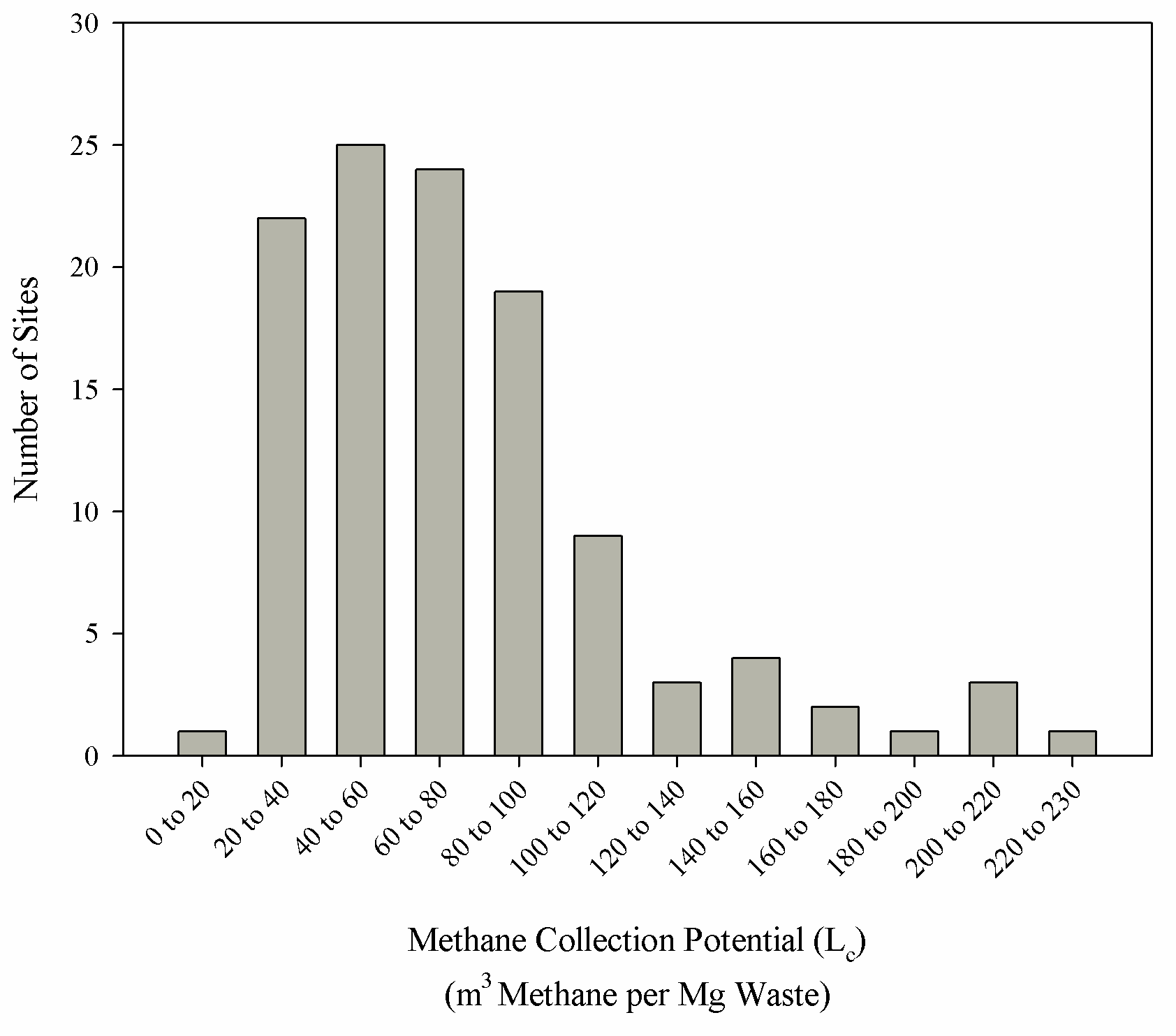

Supplement: S5 Fig — (TIF) [file pone.0246334.s005.TIF]

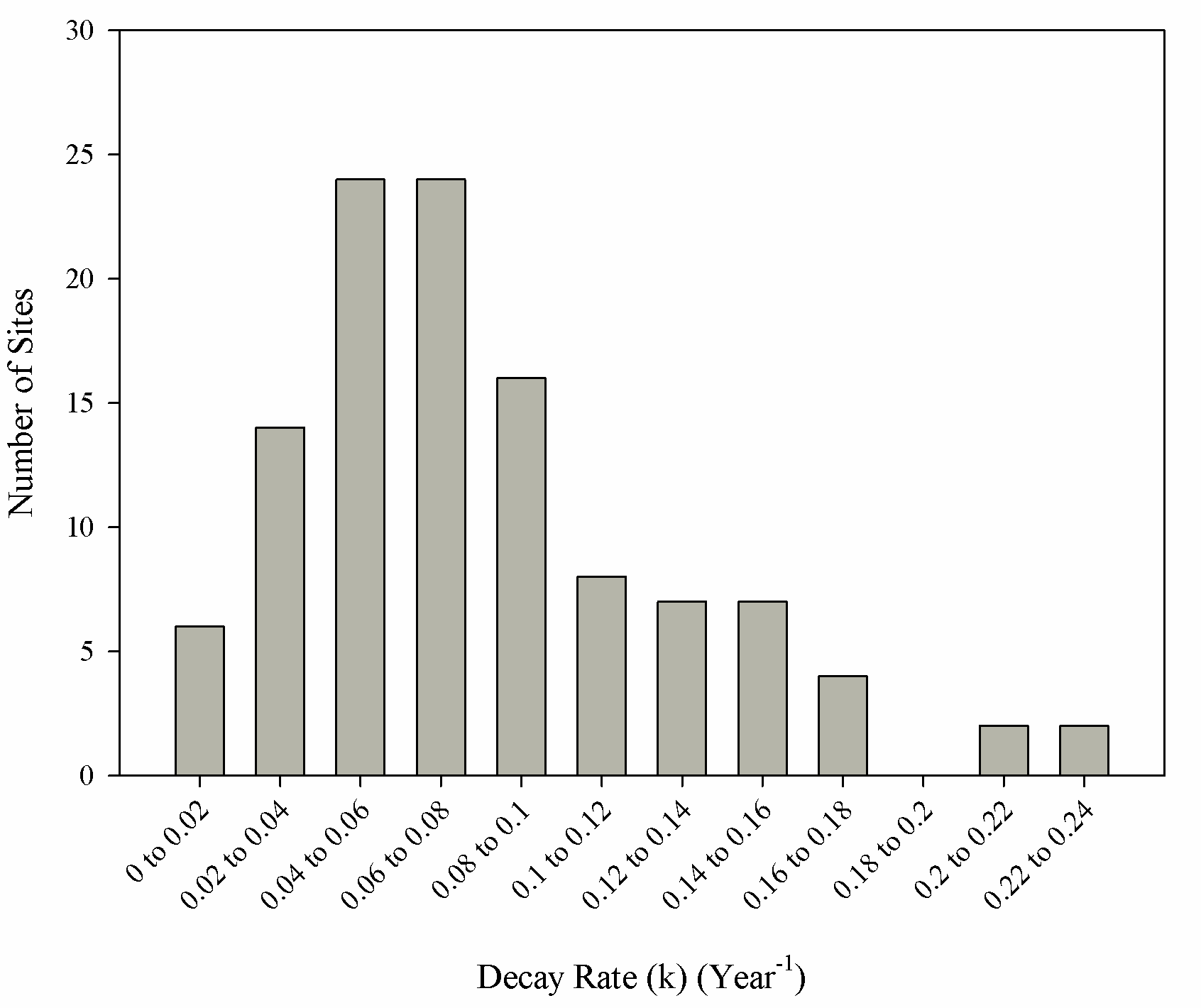

Supplement: S6 Fig — No decay rate estimates were within 0.18 to 0.2 year-1. (TIF) [file pone.0246334.s006.TIF]

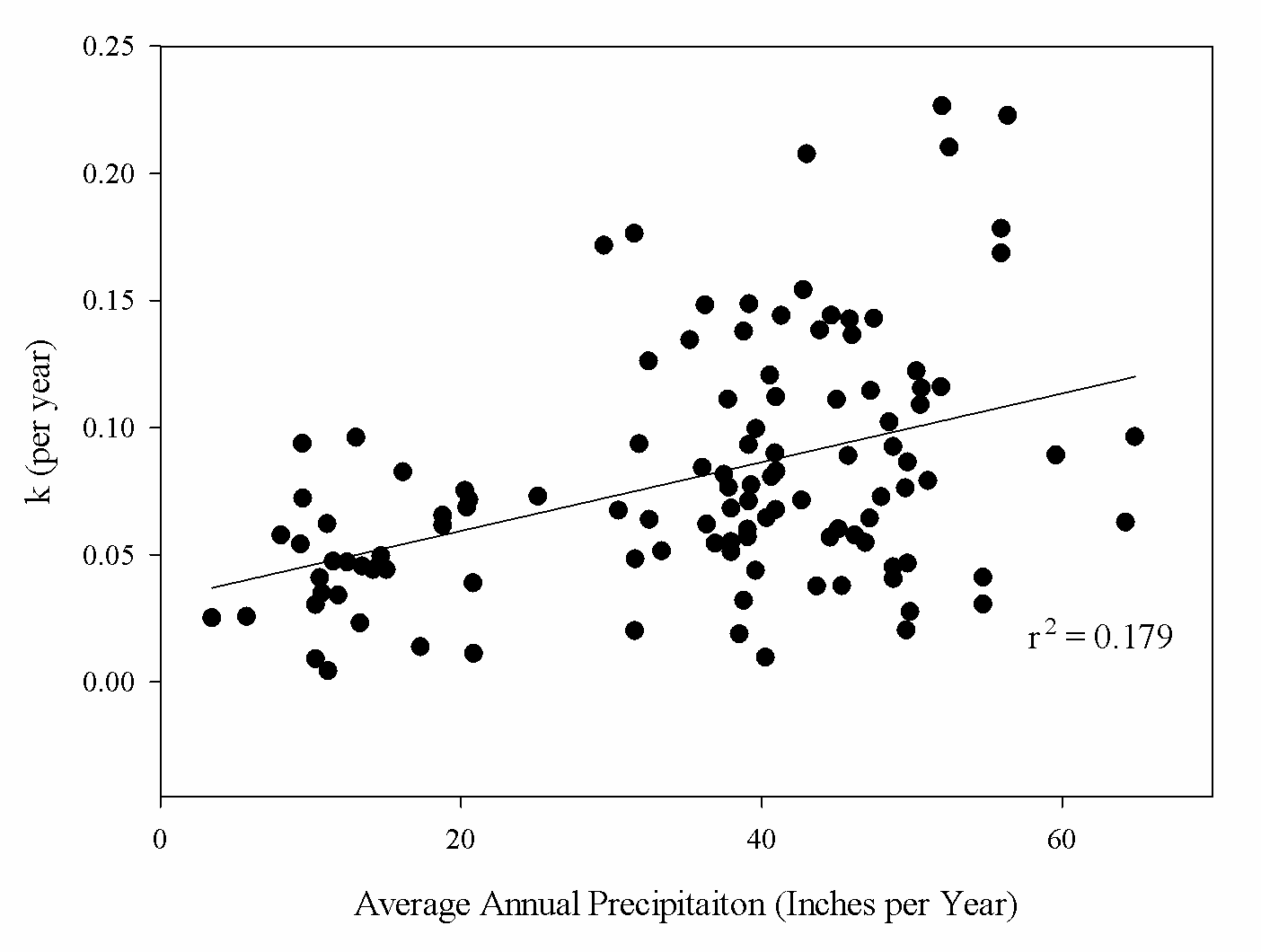

Supplement: S7 Fig — Data show some degree of correlation between precipitation and k estimates. (TIF) [file pone.0246334.s007.TIF]

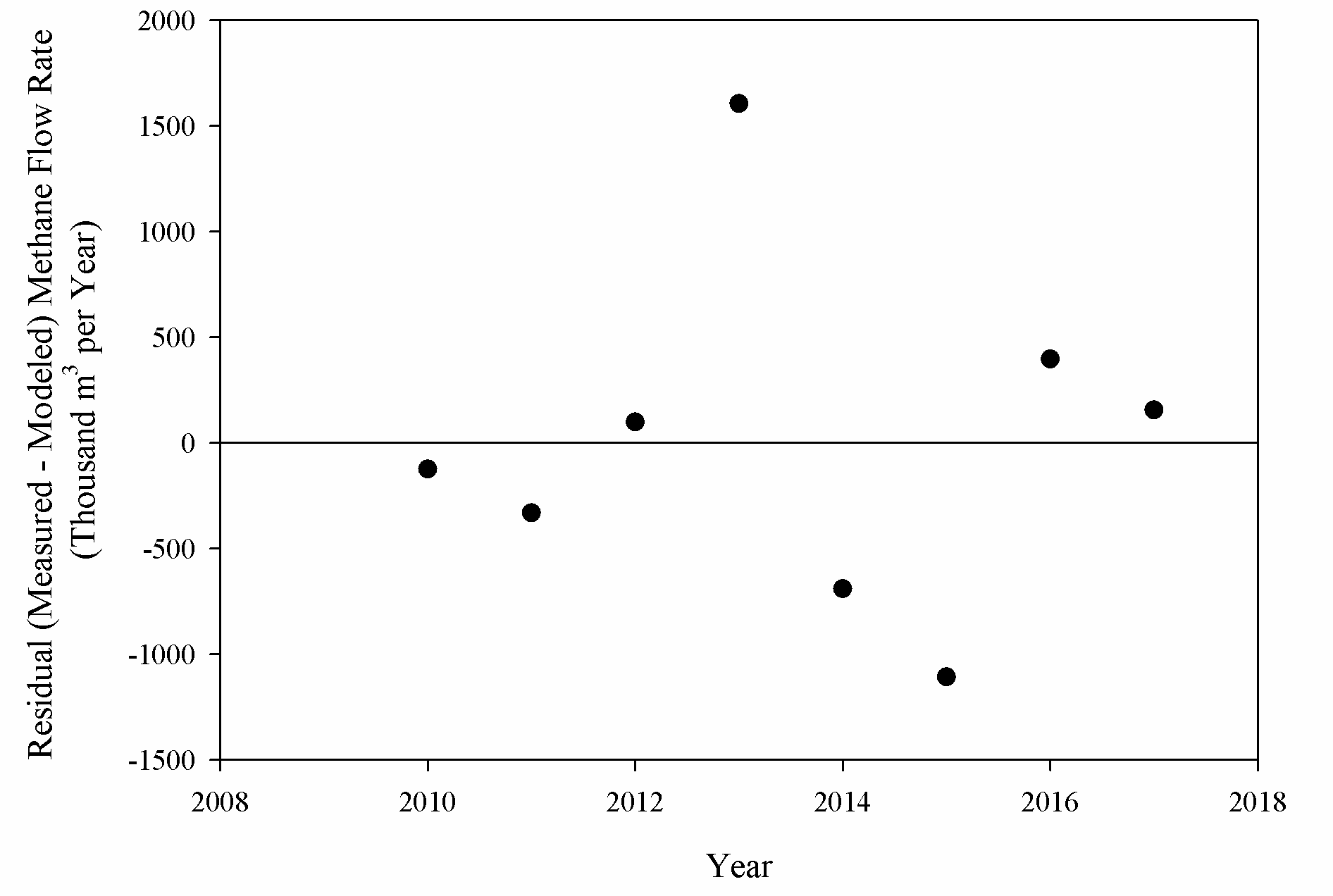

Supplement: S8 Fig — The lack of trend in the residuals and the random scatter pattern indicates a lack of bias in the modeled data. The residuals plot for each site was to confirm a lack of bias. (TIF) [file pone.0246334.s008.TIF]

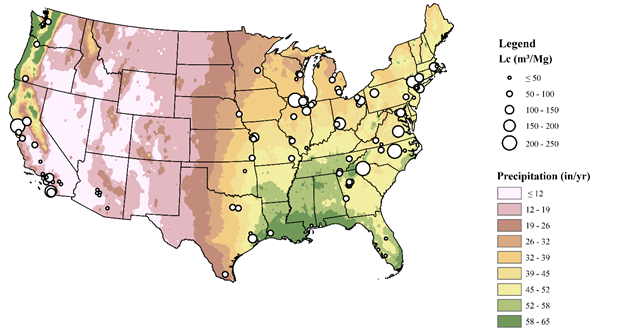

Supplement: S9 Fig — Lower precipitation regions generally had lower Lc estimate. (TIF) [file pone.0246334.s009.tif]

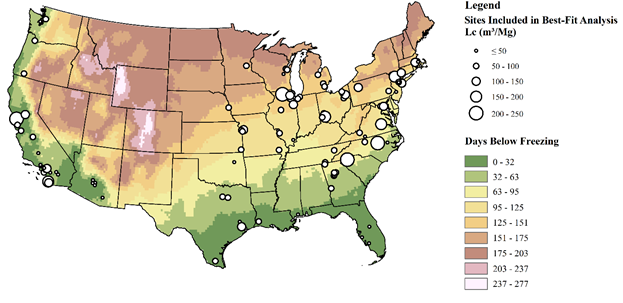

Supplement: S10 Fig — No clear trends between the days below freezing and Lc estimates were observed for the sites in the similar annual precipitation zones. (TIF) [file pone.0246334.s010.tif]

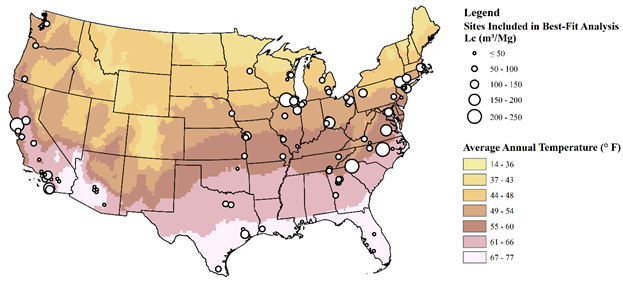

Supplement: S11 Fig — No clear trends between the average ambient annual temperature and Lc estimates were observed for the sites in the similar annual precipitation zones. (TIF) [file pone.0246334.s011.tif]

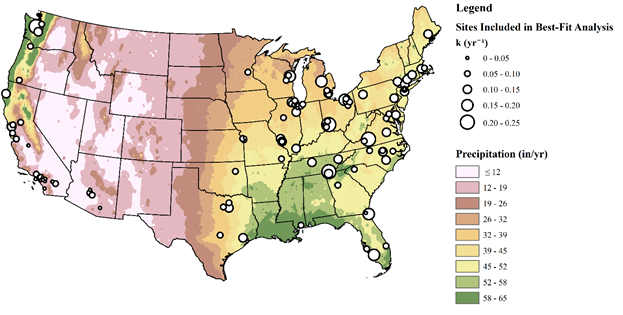

Supplement: S12 Fig — Lower precipitation regions generally had lower site-specific k estimate. (TIF) [file pone.0246334.s012.tif]

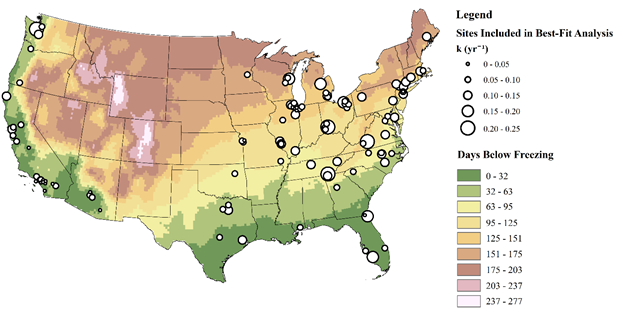

Supplement: S13 Fig — No clear trends between the days below freezing and k estimates were observed for the sites in the similar annual precipitation zones. (TIF) [file pone.0246334.s013.tif]

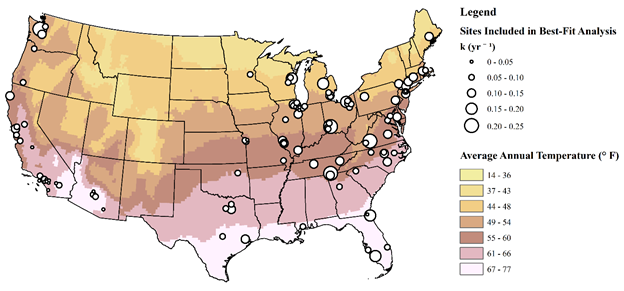

Supplement: S14 Fig — No clear trends between the average annual temperature and k estimates were observed for the sites in the similar annual precipitation zones. (TIF) [file pone.0246334.s014.tif]

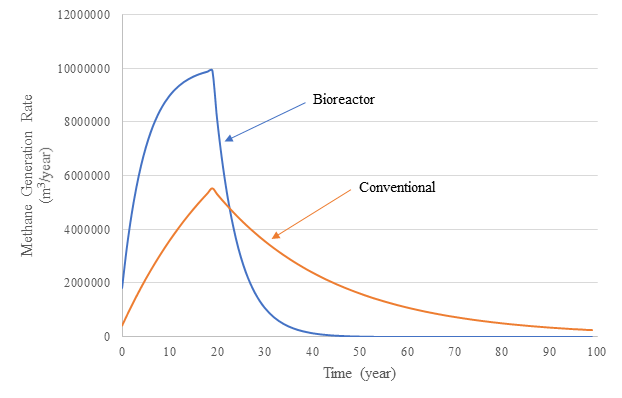

Supplement: S15 Fig — The generation rate is higher for bioreactor case before closure and lower after closure than a conventional landfill. (TIF) [file pone.0246334.s015.tif]

**Table of Contents (TOC)/Abstract Art**:

Decay Rate, k (year-1)


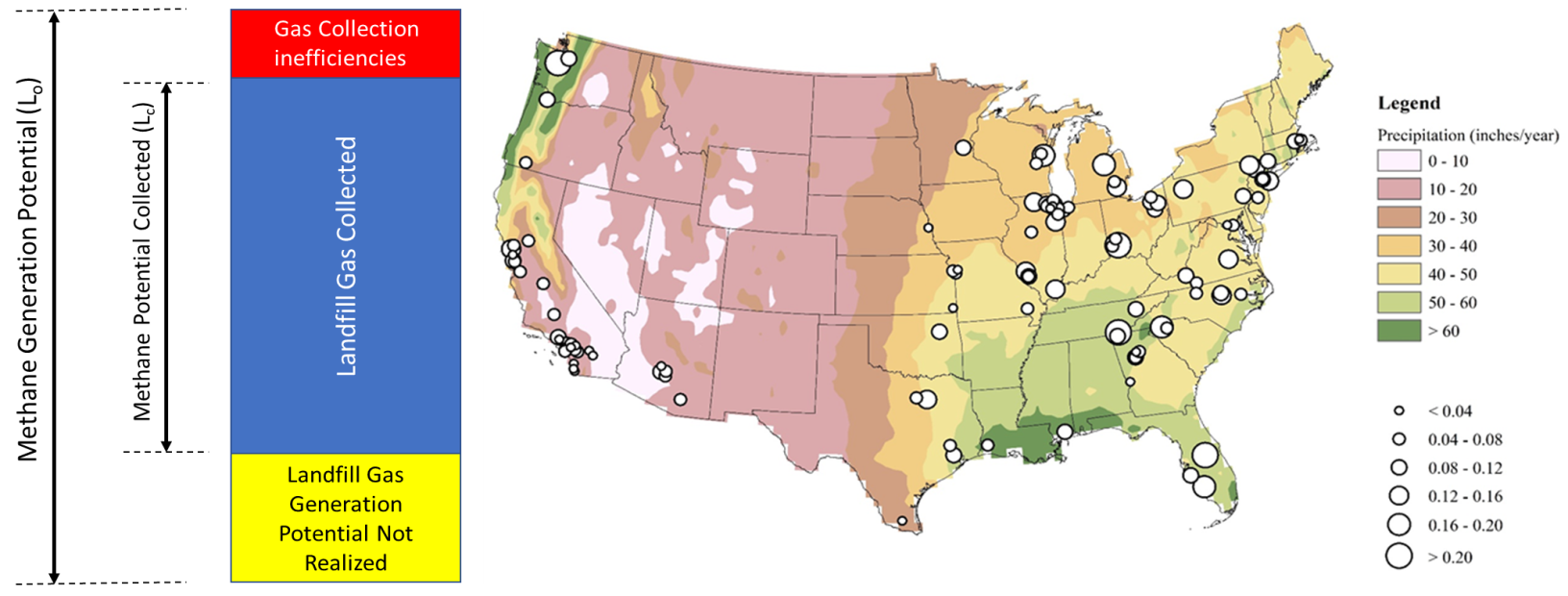

Supplement: S16 Fig — (DOCX) [file pone.0246334.s016.docx]
