# Supplementary material for: Greenhouse gas reporting data improves understanding of regional climate impact on landfill methane production and collection
Source: PLoS One. 2021 Feb 26;16(2):e0246334. doi: 10.1371/journal.pone.0246334 (PMC7909644; doi:10.1371/journal.pone.0246334)
Supplement: S1 File — (DOCX) [file pone.0246334.s017.docx]

**Supporting Information**

Mandatory Greenhouse gas reporting regulations data improves understanding of Regional climate impact on landfill methane production and collection in the US

**Pradeep Jain^1^, James Wally^1^, Timothy Townsend^2^, Max Krause^3^, Thabet Tolaymat^3*^**

^1^ Innovative Waste Consulting Services, LLC, 3720 NW 43^rd^ Street, Suite 103, Gainesville, FL 32606, USA.

^2^ Department of Environmental Engineering Sciences, University of Florida, P.O. Box 116450, Gainesville, FL 32611-6450, USA.

^3^ United States Environmental Protection Agency, Office of Research and Development, 26 W. Martin Luther King St., Cincinnati, OH 45268 USA.

*Corresponding Author: Tel.: +1 513 487 2860, fax: +1 513 569 7879; e-mail: [tolaymat.thabet@epa.gov](mailto:tolaymat.thabet@epa.gov)

**Relevant Data Included in the GHG Reporting Program Database**

The US federal regulations (40 CFR 98 Subpart HH) (referred hereinto to *GHG reporting regulations*) promulgated in 2009 require the MSW landfills owners to report annual methane generation and emissions that accepted waste on or after January 1, 1980, unless the estimated annual methane generation rate from the landfill was less than 25,000 metric tons CO_2_ equivalent. For the landfills with active gas collection and control systems, the owners are also required to report the amount of methane collected and destructed at the site. The reported data include the following data/information pertinent to the landfill and GCCS system design and operation, including the followings:

1. Operational classification (open or closed)
2. Method for estimating the historical annual waste placement amounts (e.g., direct measurement using scale(s), volumetric estimation)
3. Waste type (e.g., bulk waste, MSW, inert, etc.) and the corresponding degradable organic carbon (DOC) and a fraction of DOC dissimilated, and decay rate (k)
4. Leachate recirculation frequency for the past ten years.
5. The reported GCCS details and data include
   1. The total annual volumetric flow of landfill gas collected and destructed; the volumetric flow should be reported for the standard temperature (60 ^o^F) and pressure conditions (1 atm) (STP). Average monthly temperature and pressure should be reported if the flow is not measured under the STP conditions,
   2. Average volumetric methane concentration of the landfill gas collected and destructed,
   3. An indication of whether the flow was measured on a wet or dry basis,
   4. An indication of whether the LFG destruction occurred at on-site or off-site facilities and operating hours and efficiency of each on-site destruction device,
   5. Annual quantity of recovered methane calculated using the volumetric flow rate and methane concentration data,
   6. Description of GCCS including manufacturer, capacity, and number of wells, surface area, waste depth, and an indication of whether passive vents/flares (not connected to GCCS) are present at the site,
   7. Annual methane emission rates are estimated based on first-order decay modeling.
   8. Surface areas of landfill:
      1. Area with no waste in-place (A1)
      2. Area without active gas collection, regardless of cover type (A2)
      3. Area with daily soil cover and active gas collection (A3)
      4. Area with an intermediate soil cover, or a final soil cover not meeting the criteria for A5 below, and active gas collection (A4)
      5. Area with a final soil cover of 3 feet or thicker of clay or final cover (as approved by the relevant agency) and/or geomembrane cover system and active gas collection (A5)

US EPA publishes the reported data on a publicly accessible web portal (<https://www3.epa.gov/enviro/>). A sequential customized search was conducted on this portal to download the data reported for 2010-2017 for all the landfills that reported data over this period. All the data fields included in historical waste quantities, measurement methods, and details; gas collection system details; and landfill information tables were downloaded.

**Materials and Methods Details**

LandGEM, a landfill gas modeling tool developed and maintained by the US EPA that codifies the first order waste decay and gas generation from MSW landfills as outlined in AP-42 and the NSPS regulations, utilizes the following equation for estimating methane generation rate as a function of annual waste placement data, methane generation potential, and decay rate (US EPA 2005):

| $Q_{modeled,t}=kL_{o}\sum_{i=1}^{n} \sum_{j=0.1}^{1} \left( \frac{M_{i}}{10} \right)e^{-kt_{ij}}$ | (1) |
| --- | --- |

where Q_,modeled,t_ = annual methane generation rate in the year of the calculation (m^3^ methane per year), i = 1 year time increment, n = (year of the calculation) - (initial year of waste acceptance), j = 0.1 year time increment, k = methane generation rate (year^-1^), L_o_ = potential methane generation capacity (m^3^ methane per Mg), M_i_ = mass of waste accepted in the i^th^ year (Mg), t_ij_ = age of the j^th^ section of waste mass M_i_ accepted in the i^th^ year.

As the collection rate is expected to be lower than the generation rate due to factors such as collection system inefficiencies and occasional system shutdowns associated with power failures or routine maintenance, a collection efficiency (η) is typically applied to Eqn. 1 to calculate the methane collection rate from the generation rate estimates (Eqn. 2).

| $Q_{modeled,collected,t}=\eta kL_{o}\sum_{i=1}^{n} \sum_{j=0.1}^{1} \left( \frac{M_{i}}{10} \right)e^{-kt_{ij}}$ | (2) |
| --- | --- |

where Q_modeled,collected,t_ = annual methane collection rate in the year of the calculation (m^3^ methane per year).

A mathematically equivalent approach to estimate the methane collection rate using LandGEM is to use collection efficiency adjusted methane potential, L_c_= η L_o_. Since the analysis presented in this paper is based on the methane collection rate, the methane potential that provided the modeled methane flow rate that was the best representative of the measured flow rate is referred to herein to as the methane collection potential (L_c_) rather than the methane generation potential. The gas measurements collected at the landfill site (and corresponding modeled first-order decay estimate) more truly represent L_c_.

Substituting t_ij_ = t_ij,2009_+t in Eqn. 2, where t_ij_,_2009_ is the age of the j^th^ section of waste mass (M_i_) accepted in the i^th^ year in 2009, and t is the time since 2009, results in

$$Q_{modeled,collected,t}=\left( kL_{c}\sum_{i=1}^{n} \sum_{j=0.1}^{1} \left( \frac{M_{i}}{10} \right)e^{-kt_{ij,2009}} \right)e^{-kt}$$

 (3)

Eqn. 3 can be arranged in of the following form for simplicity,

$Q_{modeled, collected,t}=be^{-kt}$ (4)

where

$$b=kL_{c}\sum_{i=1}^{n} \sum_{j=0.1}^{1} \left( \frac{M_{i}}{10} \right)e^{-kt_{ij,2009}}$$

 (5)

The log transformation of Eqn. 4 results in a linear equation with a slope of -k and an intercept of b. The decay rate, therefore, is equivalent to the rate of change of log-transformed collected methane flow rate and is independent of the other modeling parameters (e.g., L_o_, η, waste mass, and age). The intercept b is a combination of L_o_, η, k, waste mass, and age of individual waste batches. Assuming a steady collection efficiency, the methane collection rate trend would resemble the generation rate. The k estimates are based on the collection rate data and are thus representative of waste decomposition and ensuing methane generation rates.

Excel LOGEST function, which models a power equation ($Q=bm^{t})$, was used to estimate curve fit parameters b and m that produced the best fit to the reported methane collection rates for each of 127 sites. The reported annual recovered methane quantity (metric tons per year) was converted to an annual volumetric methane collection rate (Q_measured_) (m^3^ per year) using a methane density of 0.678 kg per m^3^, which is equivalent to the GHG reporting regulation-specified methane density of 0.0423 lb per ft^3^ at 60 ^o^F and 1 atm.

The decay rate ratio and methane yield were estimated using the following equations:

$k=-\ln m$ (6)

$$L_{c}=\frac{b}{k\sum_{i=1}^{n} \sum_{j=0.1}^{1} \left( \frac{M_{i}}{10} \right)e^{-kt_{ij,2009}}}$$

 (7)

To further refine the site-specific L_c_ and k estimate, a spreadsheet model was developed for each site to estimate the annual methane generation rate using the first-order decay model (Eqn. 2) for the period with available measured methane flow data. Historical disposal data were used to model gas production for each year that the facility reported the methane collection data. The spreadsheet model was validated by comparing the methane generation rate from the model to the methane generation rates modeled from LandGEM for 15 sites. The methane generation rate estimates from the spreadsheet model were within 0.2% of those from LandGEM for all 15 sites.

The squared difference between the modeled and measured data points was calculated for every measured data point and added to calculate the sum of squared errors (SSE). The SSE is a measure of the variation between the modeled data and the measured data that is used as a measure of the relative goodness of fit for different parameter values (Eqn. 8):

$SSE=\sum_{j}^{n} \left( Q_{modeled, collected,j}-Q_{measured,j} \right)^{2}$ (8)

where Q_modeled,collected,j_ = modeled annual methane flow rate for year j, Q_measured,j_ = methane flow rate calculated using the reported annual recovered methane quantity for year j, and n = number of years annual recovered methane quantity data are reported for the site. The Excel Solver function was used to minimize the SSE between the reported annual methane collection (recovered) rate and the modeled collection rate by varying decay constant and methane generation potential values.

The site-specific k and L_c_ estimated using the MS EXCEL LOGEST function were used as initial values for SOLVER. In addition, the SOLVER function was run for three additional initial L_c_ values of 20, 100, and 230 m^3^ Mg^-1^. This range reflects the wide variation in the methane generation potential reported for MSW (Krause et al., 2016). While the initial L_c_ selection had no impact on the model solution for more than 99% of the data, in instances where different initial L_c_ values resulted in a variable model solution, the L_c_ and k values that resulted in the least SSE were selected for each site.

**Results and Discussion- Ambient Temperature Impacts on the Decay Rate (k)**

The sites were grouped by similar precipitation to control for the impact of precipitation while assessing the impact of temperature on k within each precipitation group. The study sites were classified into the following precipitation groups: <20, 20-40, and >40 inches annual precipitation) and sites within each precipitation group were further classified into four different average annual temperature sub-groups (40-50, 50-60, 60-70, and 70-80 ^o^F) and statistically compared. None of the study sites had an annual average temperature of less than 40 ^o^F or more than 80 ^o^F. The analysis was also performed by classifying the sites into two precipitation groups of more than 25 and less than 25 inches annual precipitation. No statistically significant (p<0.05) differences in k were found among different temperature sub-groups of each precipitation group. The decay rate is expected to be dependent on the in-place waste temperature. However, the waste temperature, especially in the deeper sections of the landfill, are not influenced by the ambient temperature as reported by Yessiler et al. (2005).

Table S1. Previous Studies that Used Inverse First-Order Decay Modeling to Estimate Decay Rate and/or Methane Generation Potential

| Source | Number of Landfill Sites | Location | Timeframe of the Measured Data | L_o_ (m^3^/Mg) | k (year^-1)^ |
| --- | --- | --- | --- | --- | --- |
| Pelt (1993) | 44 (14 closed; 30 active) | US | 1 (single landfill gas measurement) | Assumed/ estimated | 0.0065-0.0753 |
| Barlaz et al. (2010) | 3 (operated as a bioreactor, closure status unknown) | US and UK | 6-20 year | Assumed | 0.05-0.35 |
| Faour et al. (2007) | 6 (all closed; data from 3 cells were used for one of the sites) | North America |  | 9-115 | 0.11-2.2 |
| Amini et al. (2012) | 5 (1 closed and 4 partially closed) | FL | 3-8 years | Waste composition and first-order decay modeling | 0.04-0.13 |
| Amini et al. (2013) | 3 | US |  | Waste composition | 0.04-0.09 |
| Vu et al. (2017) | 2 (closed) | Canada | 7 years | 62-100 | 0.01-0.02 |
| Wang et al. (2013) | 11 (3 closed and 8 open) | US Northeast and Southeast Regions | > 5 years (monthly data) (3 years for one site) | Assumed to range from 55 to 100 | 0.04-0.17 |
| Zhao et al. (2013) | 1 (2 bioreactor cells) | MI | 3-4 years | Assumed | 0.082 (leachate bioreactor); 0.296 (septage bioreactor) |
| Tolaymat et al. (2010) | 1 (1 conventional cell and 2 bioreactor cells) (closed) | KY | 2-4 years (monthly data) | Lab measured | 0.06 (conventional), 0.11 (bioreactor) |
| Sun et al. (2019) | 21 (26 cells- 1 closed and 25 open) | 17 states of the US | 3-20 years | Assumed to range from 55-160 | 0.01-0.46 |

Table S2. Landfills with L_c_ values that were excluded from the analysis.

| GHGRP ID | Best-fit L_c_  (m^3^ CH_4_ per Mg waste) | Best-fit k (year^-1^) |
| --- | --- | --- |
| 1007139 | 273 | 0.277 |
| 1003723 | 3,452 | 0.360 |
| 1004541 | 320 | 0.347 |
| 1006349 | 931 | 0.206 |
| 1007947 | 288 | 0.155 |
| 1007993 | 463 | 0.094 |
| 1004741 | 301 | 0.174 |
| 1008011 | 423 | 0.040 |
| 1003503 | 259 | 0.138 |
| 1003743 | 542 | 0.145 |
| 1004653 | 353 | 0.138 |
| 1006339 | 831 | 0.225 |
| 1006390 | 281 | 0.131 |

Yes

Total number of MSWLFs = 1312

Number of MSWLFs closed before 2013= 299

Number of MSWLFs (closed before 2013) with active GCCS= 244

Yes

Are methane collection data available for at least 5 years?

Number of MSWLFs (closed before 2013) with active GCCS with at least 5 years of methane collection data = 204

Yes

No

Was gas collected from the entire site? Is landfill closed with final/ intermediate cap?

Number of Closed MSWLFs =158

Number of closed MSWLFs Considered for Further Analysis = 114

Is L_c_< 223?

Yes

Number of closed MSWLFs used for regression and inverse first-order decay modeling =127

Does annual methane collection rate increase by more than 50% or show an increasing trend?

Was it closed before 2013?

Does it have an active GCCS?

Yes

Yes

Figure S1. Flow chart depicting the site selection approach for regression and first-order decay inverse modeling

Figure S2. Example of a site that exhibited a generally increasing methane collection rate trend. Sites that exhibited an increasing trend for the methane collection rate were excluded from the study

Figure S3. Example of a site that exhibited at least one annual increase of more than 50% in methane collection rate. These sites were excluded from the analysis. There is a 74% increase from 2011 to 2012 in methane collection rate for this site.

Figure S4. Example of a methane collection rate trend of a site that was selected for regression and the first-order decay inverse modeling. The plot also includes the modeled methane collection rates corresponding to the minimum SSE. The modeled data present a good approximation of the measured methane collection rates.

Figure S5. Distribution of the site-specific methane collection potential (L_c_) values estimated based on inverse first-order decay modeling using the reported methane collection rate and waste tonnage for the 114 sites included in the study.

Figure S6. Distribution of the site-specific decay rate (k) values estimated based on inverse first-order decay modeling using the reported methane collection rate and waste tonnage for the 114 sites included in the study. No decay rate estimates were within 0.18 to 0.2 year^-1^.

Figure S7. Plot showing site-specific decay rate (k) values as a function of precipitation for the 114 study sites included in the study. Data show some degree of correlation between precipitation and k estimates.

Figure S8. Example residuals plot of a site. The lack of trend in the residuals and the random scatter pattern indicates a lack of bias in the modeled data. The residuals plot for each site was to confirm a lack of bias.


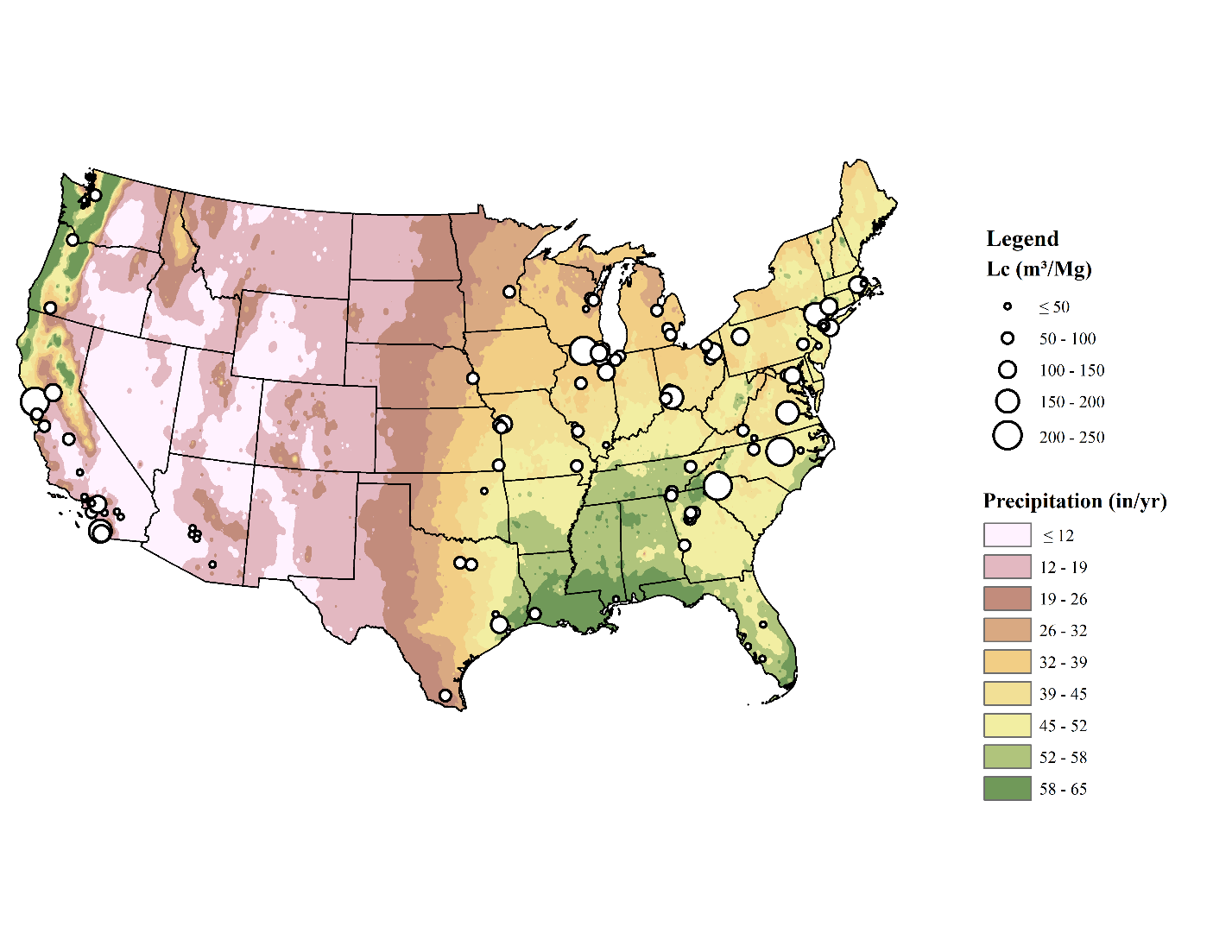


Figure S9. Location of the 114 study sites by L_c_ value compared to annual precipitation in the contiguous US. Lower precipitation regions generally had lower L_c_ estimate.


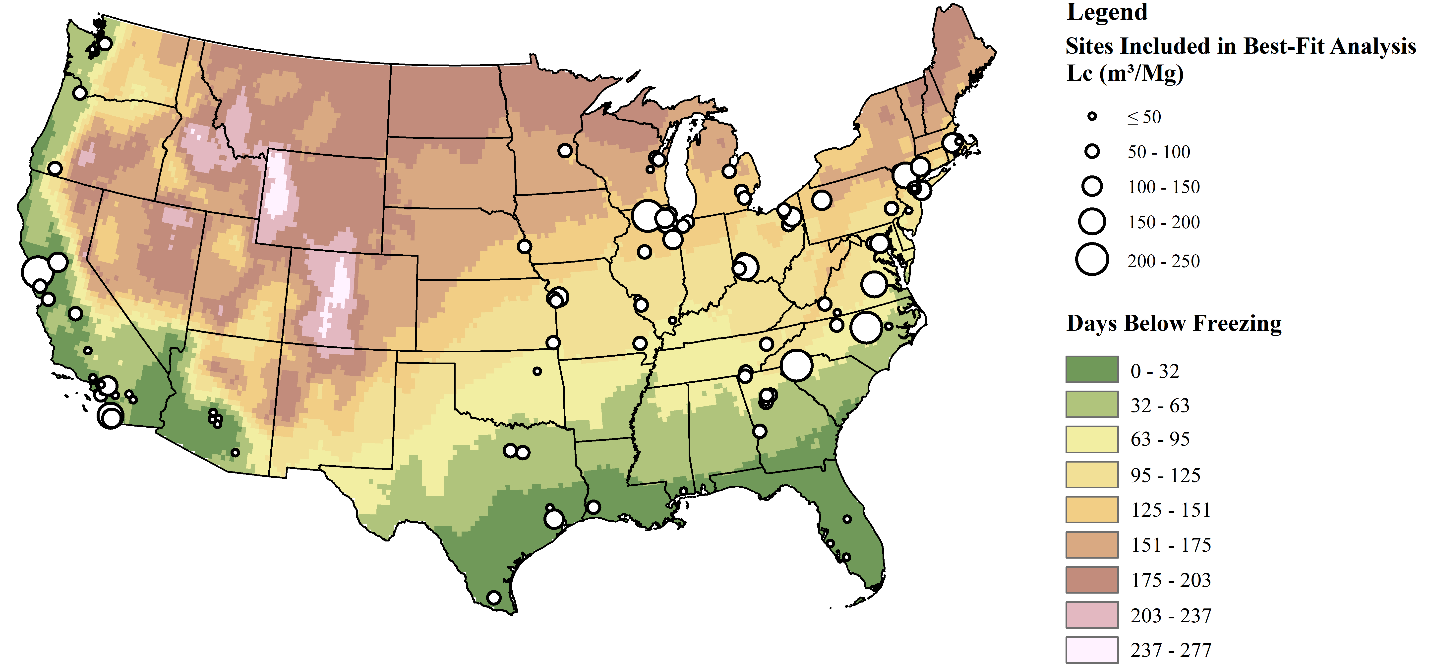


Figure S10. Location of the 114 study sites by L_c_ value compared to the number of days below freezing in the contiguous US. No clear trends between the days below freezing and L_c_ estimates were observed for the sites in the similar annual precipitation zones


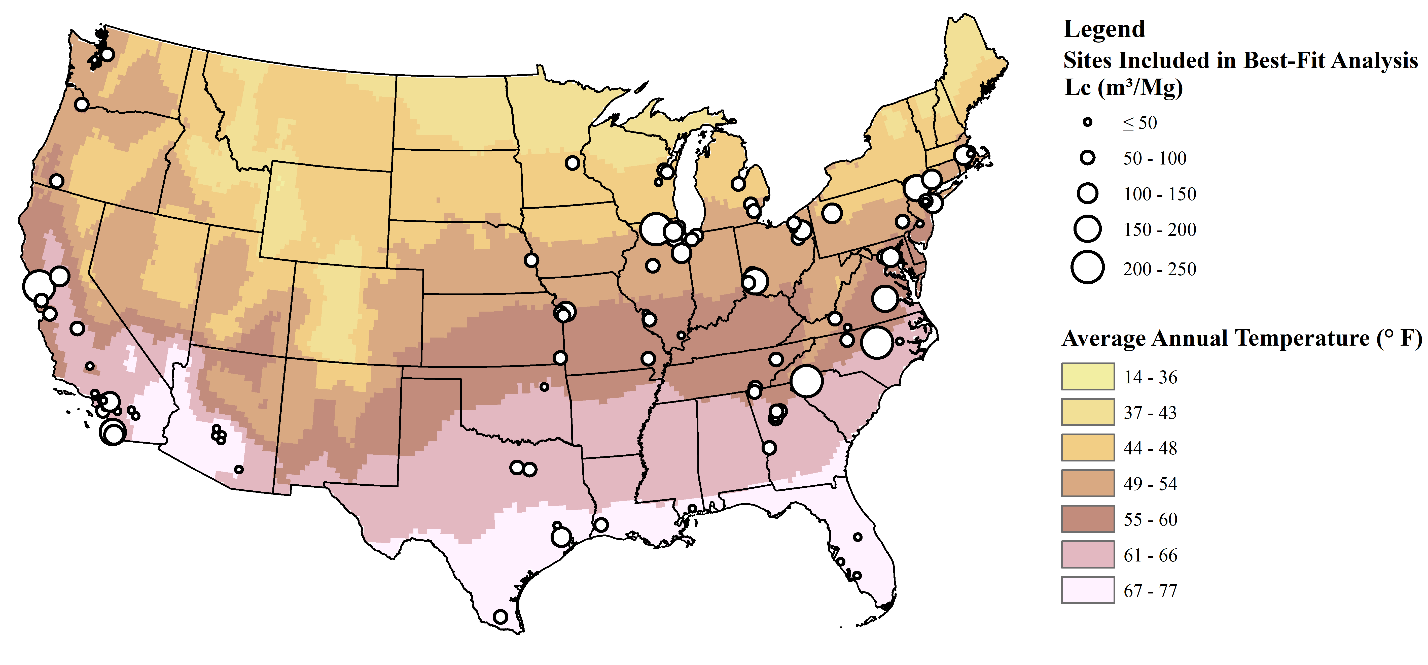


Figure S11. Location of the 114 study sites by L_c_ value compared to the site-specific average ambient temperature in the contiguous US. No clear trends between the average ambient annual temperature and L_c_ estimates were observed for the sites in the similar annual precipitation zones.


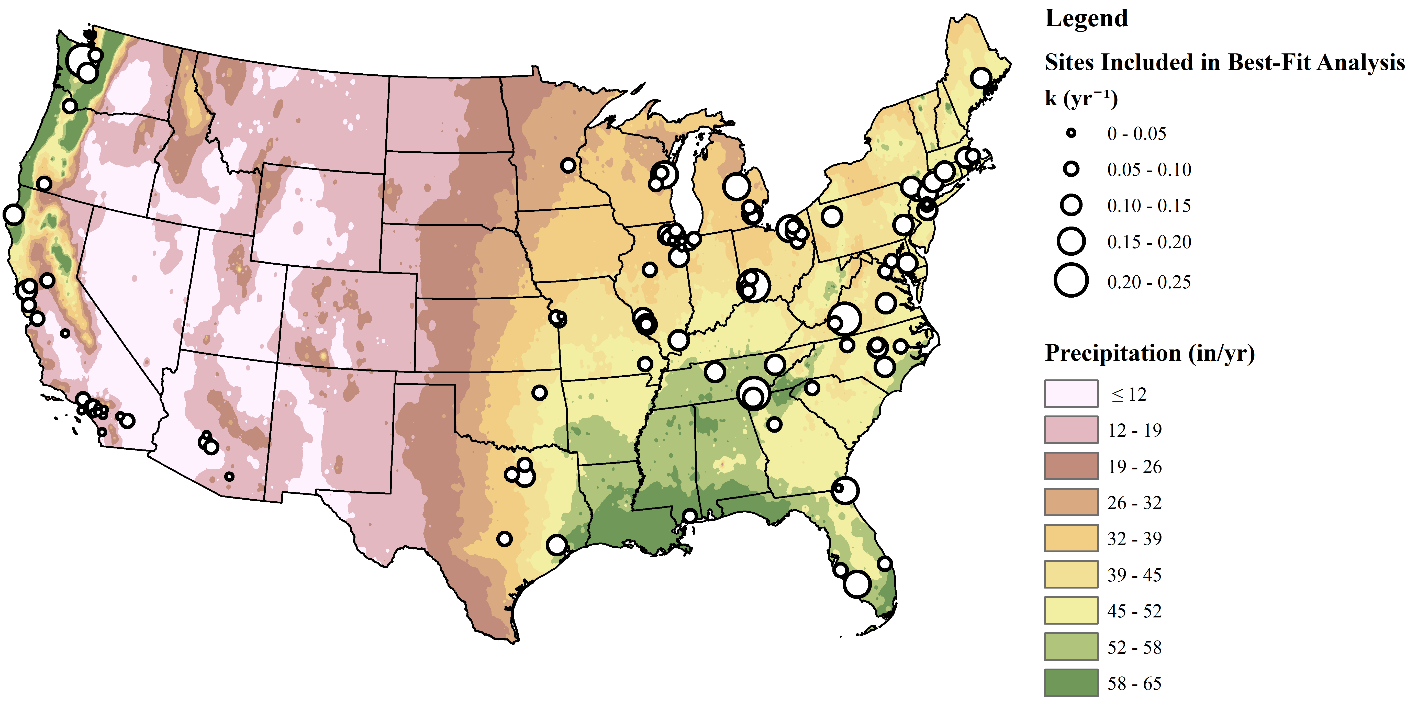


Figure S12. Location of the 114 study sites by k value compared to annual precipitation in the contiguous US. Lower precipitation regions generally had lower site-specific k estimate


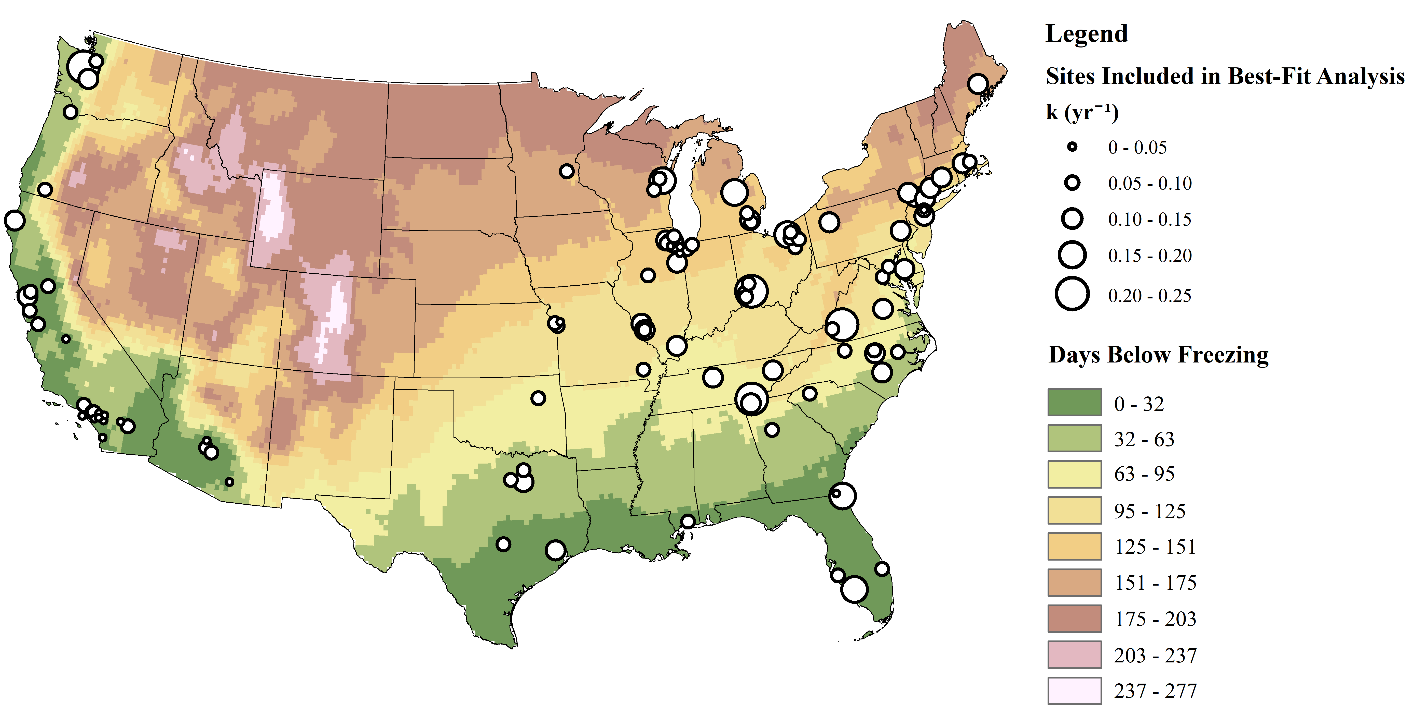


Figure S13. Location of the 114 study sites by k value compared to the number of days below freezing in the contiguous US. No clear trends between the days below freezing and k estimates were observed for the sites in the similar annual precipitation zones


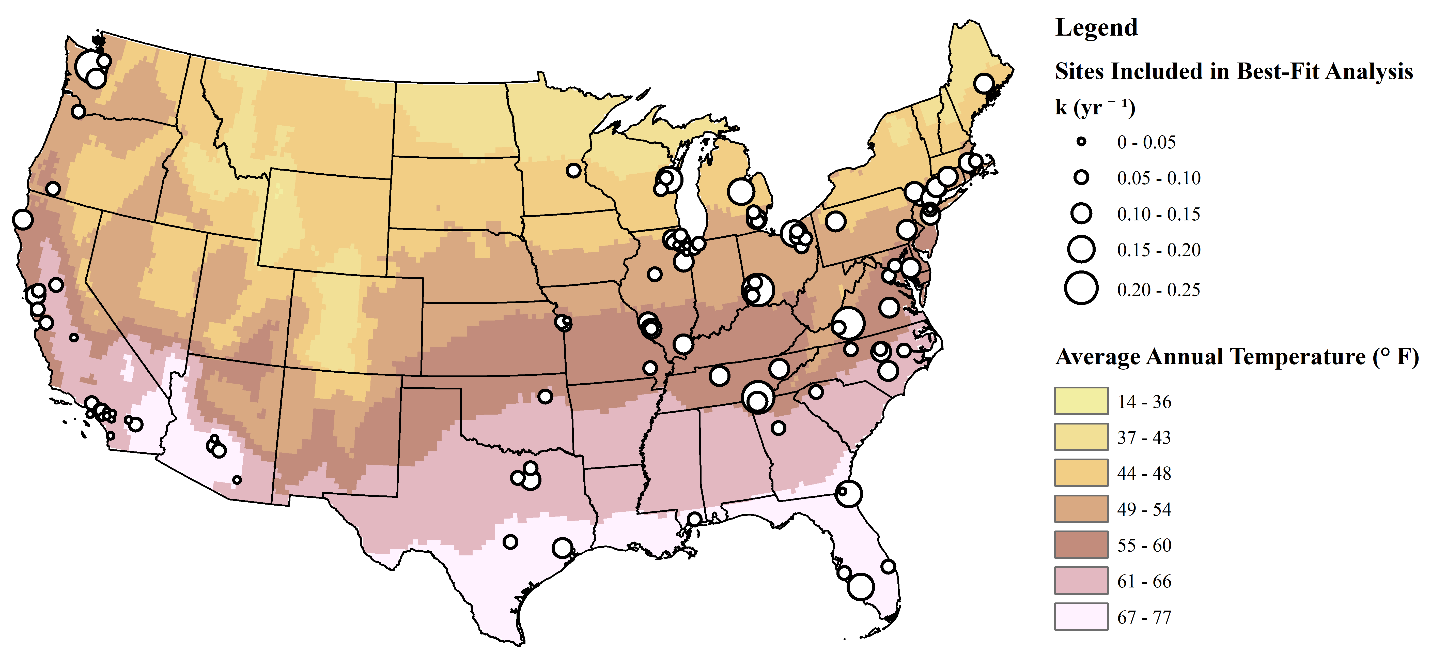


Figure S14. Location of the 114 study sites by k value compared to the average temperature in the contiguous US. No clear trends between the average annual temperature and k estimates were observed for the sites in the similar annual precipitation zones.

Conventional

Figure S15. Comparison of methane generation rate trends for a bioreactor (k=0.2 year^-1^) and conventional (k=0.04 year^-1^) landfill with an annual disposal rate of 100,000 Mg per year, a life span of 20 years and methane generation potential (L_o_) of 100 m^3^ methane per Mg waste. The generation rate is higher for bioreactor case before closure and lower after closure than a conventional landfill.

Table S3. Data used for statistical analysis for the 114 study sites. Data include location, region, timeframe of operation, k and L_c_ parameters, weather data, and leachate recirculation history.

| **Site Number** | **State** | **Total Waste in Place (Mg)** | **Opening year** | **Closure Year** | **Estimated L_c_ (m^3^/Mg)** | **Estimated k (yr^-1^)** | **Region** | **Precipitation (in/yr)** | **Days below freezing** | **Average Temp (deg F)** | **Avg Waste Placement Year** | **Years open** | **Leachate recirculation reported** |
| --- | --- | --- | --- | --- | --- | --- | --- | --- | --- | --- | --- | --- | --- |
|  |  |  |  |  |  |  |  |  |  |  |  |  |  |
| 1 | CA | 2098168 | 1974 | 1997 | 30 | 0.047 | SW | 12.44 | 35.1 | 64.6 | 1988 | 24 | N |
| 2 | CA | 1819366 | 1973 | 1994 | 37 | 0.062 | SW | 11.11 | 35.1 | 64.6 | 1983 | 22 | N |
| 3 | CA | 6644112 | 1967 | 2004 | 43 | 0.026 | SW | 5.74 | 1.2 | 73.9 | 1991 | 38 | N |
| 4 | CA | 2432511 | 1960 | 1986 | 30 | 0.023 | SW | 13.29 | 2.5 | 65.8 | 1973 | 27 | N |
| 5 | NE | 4738966 | 1973 | 1989 | 95 | 0.020 | NE | 31.56 | 145.6 | 49.6 | 1982 | 17 | N |
| 6 | NY | 4705000 | 1961 | 1991 | 150 | 0.154 | NE | 42.77 | 95.3 | 54.5 | 1976 | 31 | N |
| 7 | CA | 35374149 | 1963 | 1990 | 71 | 0.041 | SW | 10.62 | 0 | 61.9 | 1976 | 28 | N |
| 8 | CA | 12267058 | 1968 | 1998 | 37 | 0.045 | SW | 13.43 | 0 | 65.3 | 1983 | 31 | N |
| 9 | OR | 9203618 | 1960 | 1991 | 55 | 0.093 | NW | 39.14 | 17.7 | 54.1 | 1979 | 32 | N |
| 10 | AZ | 2045082 | 1972 | 1997 | 32 | 0.044 | SW | 14.15 | 23.6 | 68.7 | 1985 | 26 | N |
| 11 | IL | 17742074 | 1968 | 2006 | 65 | 0.055 | NE | 36.91 | 121 | 49.9 | 1992 | 39 | N |
| 12 | CA | 3255310 | 1983 | 1992 | 19 | 0.058 | SW | 8.03 | 20 | 64 | 1987 | 10 | N |
| 13 | FL | 5352693 | 1971 | 1998 | 46 | 0.089 | SE | 59.57 | 3.4 | 73.1 | 1988 | 28 | N |
| 14 | AZ | 10432611 | 1973 | 1993 | 28 | 0.054 | SW | 9.33 | 12.5 | 71.3 | 1983 | 21 | N |
| 15 | NY | 2533926 | 1972 | 2009 | 77 | 0.122 | NE | 50.29 | 167.4 | 46.4 | 1998 | 38 | N |
| 16 | OH | 4383991 | 1979 | 1993 | 77 | 0.065 | NE | 40.33 | 101.9 | 54.1 | 1985 | 15 | N |
| 17 | CA | 15662897 | 1957 | 2000 | 72 | 0.062 | SW | 18.79 | 1.9 | 65 | 1986 | 44 | N |
| 18 | MD | 6247986 | 1965 | 1982 | 83 | 0.010 | NE | 40.26 | 74.3 | 55.7 | 1973 | 18 | N |
| 19 | GA | 2330969 | 1963 | 2000 | 55 | 0.041 | SE | 54.73 | 62.9 | 61.7 | 1981 | 38 | N |
| 20 | IL | 5410547 | 1973 | 2004 | 34 | 0.144 | NE | 41.3 | 83 | 56.7 | 1993 | 32 | N |
| 21 | PA | 11200836 | 1948 | 2005 | 82 | 0.114 | NE | 47.26 | 83.5 | 52.6 | 1977 | 58 | N |
| 22 | WA | 13253915 | 1963 | 2003 | 34 | 0.223 | NW | 56.37 | 37.4 | 52 | 1983 | 41 | N |
| 23 | IL | 14318815 | 1970 | 1993 | 86 | 0.051 | NE | 33.35 | 115.3 | 50.6 | 1982 | 24 | Y |
| 24 | IL | 8691568 | 1960 | 1982 | 99 | 0.032 | NE | 38.82 | 142 | 48.8 | 1972 | 23 | N |
| 25 | IL | 4451937 | 1971 | 1997 | 91 | 0.044 | NE | 39.6 | 123.1 | 50.1 | 1989 | 27 | N |
| 26 | CA | 7076024 | 1942 | 1980 | 43 | 0.039 | NW | 20.81 | 2.9 | 57.5 | 1961 | 39 | N |
| 27 | OH | 4300034 | 1940 | 1990 | 99 | 0.100 | NE | 39.62 | 119.3 | 49.9 | 1965 | 51 | N |
| 28 | TX | 2325840 | 1979 | 1998 | 119 | 0.116 | SE | 50.63 | 5.1 | 70.7 | 1989 | 20 | N |
| 29 | MO | 3135654 | 1969 | 1997 | 125 | 0.038 | NE | 43.67 | 109.6 | 54.5 | 1983 | 29 | N |
| 30 | GA | 2113662 | 1983 | 1998 | 69 | 0.020 | SE | 49.62 | 41.4 | 64.4 | 1990 | 16 | N |
| 31 | SC | 4838583 | 1991 | 2007 | 44 | 0.064 | SE | 47.19 | 52.1 | 61.3 | 2000 | 17 | Y |
| 32 | SC | 2274520 | 1972 | 1987 | 221 | 0.178 | SE | 55.94 | 54.1 | 60.5 | 1981 | 16 | N |
| 33 | AZ | 14626219 | 1973 | 2005 | 26 | 0.035 | SW | 10.76 | 1.5 | 72.1 | 1994 | 33 | N |
| 34 | KS | 3989935 | 1985 | 2006 | 62 | 0.057 | NE | 39.06 | 89.9 | 56.7 | 1996 | 22 | N |
| 35 | MI | 5987419 | 1981 | 1993 | 84 | 0.064 | NE | 32.53 | 145.1 | 47.2 | 1987 | 13 | N |
| 36 | IL | 3847728 | 1960 | 1997 | 57 | 0.068 | NE | 40.94 | 99.4 | 55.7 | 1979 | 38 | N |
| 37 | MO | 5987421 | 1972 | 2002 | 79 | 0.060 | NE | 39.06 | 89.9 | 54.8 | 1987 | 31 | N |
| 38 | CA | 4990034 | 1962 | 1996 | 28 | 0.050 | NW | 14.68 | 1.3 | 60.3 | 1979 | 35 | N |
| 39 | KS | 2609032 | 1976 | 2010 | 95 | 0.038 | NE | 45.34 | 87.5 | 57.4 | 1993 | 35 | Y |
| 40 | IL | 2125535 | 1974 | 1993 | 72 | 0.112 | NE | 40.94 | 95.2 | 57.3 | 1984 | 20 | N |
| 41 | OH | 8762077 | 1960 | 1999 | 173 | 0.208 | NE | 43 | 116.4 | 53.4 | 1980 | 40 | N |
| 42 | MO | 2119950 | 1980 | 2010 | 60 | 0.073 | NE | 47.95 | 92.1 | 58.4 | 1995 | 31 | N |
| 43 | CA | 4380976 | 1966 | 1995 | 49 | 0.069 | NW | 20.39 | 12.2 | 56.4 | 1981 | 30 | N |
| 44 | OH | 2054665 | 1971 | 1987 | 78 | 0.071 | NE | 42.66 | 113 | 53.3 | 1979 | 17 | N |
| 45 | MA | 3841927 | 1973 | 1996 | 80 | 0.028 | NE | 49.88 | 137.5 | 49.2 | 1985 | 24 | N |
| 46 | MA | 6735847 | 1975 | 1998 | 117 | 0.109 | NE | 50.56 | 141.8 | 49.2 | 1987 | 24 | N |
| 47 | OH | 9036204 | 1961 | 1993 | 117 | 0.081 | NE | 40.66 | 152.2 | 47.7 | 1977 | 33 | N |
| 48 | GA | 4367186 | 1993 | 2011 | 86 | 0.047 | SE | 49.71 | 36.3 | 62.6 | 2001 | 19 | N |
| 49 | LA | 1147589 | 1960 | 1993 | 52 | 0.063 | SE | 64.21 | 10.9 | 68.6 | 1977 | 34 | Y |
| 50 | TX | 4490565 | 1979 | 1995 | 80 | 0.076 | SE | 37.8 | 32.3 | 65.8 | 1987 | 17 | N |
| 51 | VA | 1578501 | 1972 | 2005 | 49 | 0.060 | NE | 45.12 | 115.3 | 55.5 | 1993 | 34 | N |
| 52 | IN | 9617057 | 1979 | 2002 | 59 | 0.071 | NE | 39.14 | 116.5 | 50.3 | 1992 | 24 | N |
| 53 | TN | 1672754 | 1982 | 1993 | 83 | 0.116 | SE | 51.93 | 84.5 | 57.8 | 1988 | 12 | N |
| 54 | TX | 5538365 | 1976 | 2002 | 88 | 0.121 | SE | 40.55 | 44 | 65.1 | 1989 | 27 | N |
| 55 | WI | 2730000 | 1977 | 1996 | 56 | 0.068 | NE | 30.49 | 154.5 | 44.9 | 1987 | 20 | N |
| 56 | VA | 1894280 | 1981 | 1993 | 157 | 0.144 | NE | 44.64 | 116.9 | 56 | 1988 | 13 | N |
| 57 | NY | 5394000 | 1963 | 2002 | 167 | 0.138 | NE | 43.87 | 116.3 | 51.2 | 1984 | 40 | N |
| 58 | WI | 3206537 | 1979 | 2003 | 69 | 0.172 | NE | 29.52 | 156.8 | 44.3 | 1991 | 25 | Y |
| 59 | CA | 2072910 | 1964 | 2007 | 62 | 0.072 | NW | 20.54 | 18.3 | 60.7 | 1990 | 44 | Y |
| 60 | FL | 1185000 | 1986 | 2004 | 32 | 0.226 | SE | 52 | 4.3 | 71.8 | 1996 | 19 | N |
| 61 | VA | 1123231 | 1979 | 2000 | 61 | 0.090 | NE | 40.89 | 129.2 | 51.6 | 1990 | 22 | N |
| 62 | OR | 1406210 | 1960 | 1998 | 89 | 0.073 | NW | 25.12 | 85.9 | 52.8 | 1988 | 39 | N |
| 63 | CA | 5341554 | 1960 | 2006 | 203 | 0.134 | NW | 35.23 | 0.5 | 58.5 | 1983 | 47 | N |
| 64 | MI | 7832199 | 1975 | 1993 | 79 | 0.126 | NE | 32.48 | 139.4 | 48.5 | 1984 | 19 | N |
| 65 | CA | 2937316 | 1972 | 1997 | 45 | 0.025 | SW | 3.44 | 1.6 | 75.8 | 1985 | 26 | N |
| 66 | NC | 5518402 | 1986 | 2008 | 67 | 0.089 | SE | 45.76 | 79.8 | 59.1 | 2000 | 23 | N |
| 67 | MI | 4411367 | 1982 | 1999 | 60 | 0.176 | NE | 31.54 | 150.2 | 47.1 | 1991 | 18 | N |
| 68 | TX | 3200645 | 1988 | 2003 | 64 | 0.011 | SE | 20.85 | 2.8 | 74.3 | 1996 | 16 | N |
| 69 | CA | 11543810 | 1968 | 1993 | 88 | 0.083 | NW | 16.15 | 1.3 | 60.3 | 1978 | 26 | N |
| 70 | CA | 3171902 | 1960 | 1998 | 37 | 0.030 | SW | 10.34 | 2.1 | 65.5 | 1986 | 39 | N |
| 71 | OH | 6142376 | 1970 | 2002 | 33 | 0.082 | NE | 37.5 | 98.6 | 51.9 | 1986 | 33 | N |
| 72 | CA | 15033347 | 1957 | 1985 | 38 | 0.014 | SW | 17.31 | 2.2 | 64.1 | 1971 | 29 | N |
| 73 | MS | 2282105 | 1979 | 1997 | 42 | 0.096 | SE | 64.83 | 10.7 | 68.4 | 1988 | 19 | N |
| 74 | IL | 6078393 | 1975 | 2007 | 64 | 0.051 | NE | 37.98 | 115.9 | 51.9 | 1991 | 33 | N |
| 75 | IL | 14768484 | 1974 | 1996 | 130 | 0.019 | NE | 38.52 | 142 | 48.8 | 1985 | 23 | N |
| 76 | CA | 11702683 | 1979 | 1997 | 51 | 0.034 | SW | 11.84 | 0 | 61.8 | 1988 | 19 | N |
| 77 | GA | 15188575 | 1986 | 2004 | 45 | 0.086 | SE | 49.71 | 36.3 | 62.6 | 1996 | 19 | N |
| 78 | AZ | 9730034 | 1978 | 1995 | 42 | 0.094 | SW | 9.46 | 8.3 | 75.1 | 1986 | 18 | N |
| 79 | TN | 5835828 | 1980 | 1999 | 77 | 0.210 | SE | 52.48 | 58.3 | 60.9 | 1991 | 20 | N |
| 80 | IL | 8037745 | 1980 | 2008 | 64 | 0.138 | NE | 38.82 | 108.3 | 49.9 | 1994 | 29 | N |
| 81 | CA | 4714816 | 1957 | 1999 | 133 | 0.044 | SW | 15.04 | 1 | 65.5 | 1978 | 43 | N |
| 82 | IL | 9707178 | 1976 | 2002 | 116 | 0.111 | NE | 37.76 | 137.9 | 49.9 | 1989 | 27 | N |
| 83 | IL | 3026340 | 1976 | 2005 | 101 | 0.149 | NE | 39.16 | 119.4 | 50.9 | 1991 | 30 | N |
| 84 | WA | 3522190 | 1980 | 1992 | 79 | 0.084 | NW | 36.05 | 49.4 | 51.6 | 1986 | 13 | N |
| 85 | CA | 3538020 | 1968 | 1994 | 104 | 0.075 | NW | 20.27 | 5.1 | 62.7 | 1981 | 27 | N |
| 86 | IL | 1995806 | 1976 | 1995 | 218 | 0.148 | NE | 36.24 | 143.9 | 49.2 | 1986 | 20 | N |
| 87 | CA | 4241089 | 1967 | 1996 | 62 | 0.047 | NW | 11.5 | 10.5 | 64.4 | 1982 | 30 | N |
| 88 | IL | 2999900 | 1977 | 1998 | 39 | 0.055 | NE | 37.96 | 93.3 | 52.7 | 1991 | 22 | Y |
| 89 | WI | 3664368 | 1968 | 1989 | 21 | 0.048 | NE | 31.59 | 142.8 | 46.4 | 1979 | 22 | N |
| 90 | NJ | 14758314 | 1968 | 1986 | 22 | 0.058 | NE | 46.21 | 109.3 | 54 | 1977 | 19 | Y |
| 91 | AZ | 2023022 | 1981 | 2005 | 35 | 0.072 | SW | 9.49 | 8.6 | 70.8 | 1993 | 25 | N |
| 92 | NC | 4203298 | 1990 | 2004 | 67 | 0.055 | SE | 46.9 | 65.3 | 59.1 | 1997 | 15 | N |
| 93 | GA | 1470908 | 1973 | 2004 | 60 | 0.111 | SE | 44.99 | 83.8 | 59.2 | 1990 | 32 | N |
| 94 | MA | 2794129 | 1977 | 1992 | 45 | 0.079 | NE | 51.06 | 145.3 | 49.2 | 1985 | 16 | N |
| 95 | CA | 6259577 | 1973 | 1983 | 186 | 0.004 | SW | 11.16 | 0.3 | 63.1 | 1978 | 11 | N |
| 96 | MD | 7439097 | 1978 | 2000 | 106 | 0.057 | NE | 44.56 | 109 | 54.7 | 1989 | 23 | N |
| 97 | TX | 3341958 | 1975 | 1996 | 35 | 0.041 | SE | 48.77 | 17.6 | 67.4 | 1986 | 22 | N |
| 98 | IL | 20347621 | 1975 | 1999 | 101 | 0.068 | NE | 37.97 | 161.5 | 48 | 1987 | 25 | N |
| 99 | IN | 2836860 | 1971 | 1993 | 92 | 0.077 | NE | 39.3 | 126.3 | 49.9 | 1982 | 23 | N |
| 100 | OK | 4468880 | 1977 | 2003 | 47 | 0.083 | SE | 40.97 | 84.9 | 59.6 | 1990 | 27 | N |
| 101 | GA | 4354470 | 1955 | 1999 | 86 | 0.031 | SE | 54.73 | 73.9 | 61.7 | 1977 | 45 | N |
| 102 | OH | 9117207 | 1971 | 1996 | 93 | 0.062 | NE | 36.35 | 89.9 | 52.7 | 1989 | 26 | N |
| 103 | IL | 1089211 | 1983 | 2006 | 42 | 0.143 | NE | 47.46 | 96.7 | 56.5 | 1995 | 24 | Y |
| 104 | CA | 57964832 | 1958 | 2007 | 40 | 0.096 | SW | 13.02 | 2.2 | 64.1 | 1982 | 50 | N |
| 105 | PA | 3420569 | 1989 | 2008 | 102 | 0.143 | NE | 45.86 | 149.5 | 47.2 | 1999 | 20 | N |
| 106 | FL | 7388182 | 1974 | 2009 | 24 | 0.169 | SE | 55.93 | 0.5 | 74.4 | 1993 | 36 | N |
| 107 | CT | 5481214 | 1962 | 1995 | 145 | 0.102 | NE | 48.49 | 143 | 49.4 | 1979 | 34 | N |
| 108 | CA | 3058091 | 1960 | 1981 | 150 | 0.009 | SW | 10.34 | 0.1 | 65.5 | 1971 | 22 | N |
| 109 | NJ | 8800600 | 1960 | 1988 | 64 | 0.045 | NE | 48.75 | 90.3 | 54.8 | 1974 | 29 | N |
| 110 | NJ | 45886307 | 1960 | 2005 | 37 | 0.092 | NE | 48.75 | 90.3 | 54.8 | 1977 | 46 | N |
| 111 | NC | 2959866 | 1974 | 1995 | 44 | 0.076 | SE | 49.56 | 56 | 61.7 | 1984 | 22 | N |
| 112 | NC | 4345874 | 1972 | 1997 | 208 | 0.137 | SE | 46.02 | 61.1 | 60.6 | 1985 | 26 | N |
| 113 | CA | 44667000 | 1963 | 1996 | 47 | 0.065 | SW | 18.79 | 1.9 | 65 | 1987 | 34 | N |
| 114 | MN | 2899719 | 1967 | 1993 | 52 | 0.094 | NE | 31.86 | 171 | 43.5 | 1980 | 27 | N |

Table S4. Annual methane collection data reported for the 114 study sites for each year of GHGDB reporting. Measured data included in this table were used for regression and inverse first-order decay modeling.

| **Site Number** | **Reported Annual Methane Collection Rate** | | | | | | | |
| --- | --- | --- | --- | --- | --- | --- | --- | --- |
|  | **2010** | **2011** | **2012** | **2013** | **2014** | **2015** | **2016** | **2017** |
| 1 | 790.38 | 736.27 | 733.66 | 679.53 | 649.46 |  |  |  |
| 2 | 588.71 | 574.54 | 497.67 | 488.38 | 470.65 |  |  |  |
| 3 | 3295.64 | 3066.79 | 2993.76 | 2918.74 | 2879.74 | 2890 |  |  |
| 4 | 499.17 | 483.03 | 456.76 | 457.88 | 457.88 |  |  |  |
| 5 | 3784.53 |  | 3444.56 | 3270.47 | 2894 | 3265.68 | 3174.51 | 3380.2 |
| 6 | 910.1 | 796.68 | 658.18 | 800.12 | 663.28 | 465.51 | 108.21 |  |
| 7 | 15619.27 | 19348.49 | 18463.57 | 18288.83 | 16365.39 | 15674.52 | 15181.91 | 11432.88 |
| 8 | 4725.98 | 4409.31 | 3863.34 | 3566.23 | 3568.74 | 4001.68 | 3422.12 | 3327.31 |
| 9 | 2632 | 2268 | 1947 | 1718.83 | 1766 | 1745.49 | 1511.3 | 1234.18 |
| 10 | 680.7 | 619.7 | 695.9 | 578.9 | 560.33 |  |  |  |
| 11 | 19937.75 | 19620 | 15463.63 | 17306.9 | 15518.6 | 14977.61 | 14527.22 | 13516.44 |
| 12 | 663.73 | 630.51 | 646.18 | 564 | 518.89 |  |  |  |
| 13 | 2708.16 | 2284.52 | 2071.35 | 1902.54 | 1576.3 | 1386.51 | 1769.5 | 1517.2 |
| 14 | 2537.2 | 2868.5 | 2156.5 | 2784.51 | 1886.1 |  |  |  |
| 15 | 4704 | 4566.4 | 4251 | 3544 | 2921.02 | 2658.35 | 2266.54 |  |
| 16 | 3104.691 | 3080 | 3090.323 | 2664.53 | 2425.61 | 2297.47 |  |  |
| 17 | 13211.39 | 12298.92 | 11729 | 10468 | 9947.73 | 10256.27 | 8905 | 8534.8 |
| 18 | 2291.65 | 2474.88 | 2298.2 | 2386.8 | 2221.18 |  |  |  |
| 19 | 1132.3 | 1235.25 | 1196.5 | 1117.83 | 947.49 |  |  |  |
| 20 | 2402.641 | 1962 | 1857.378 | 1427.5 | 1298.24 | 1096.76 | 992.41 | 964.48 |
| 21 | 7169.72 | 5459 | 5606.189 | 5056.94 | 4531.49 | 3627.44 | 3346.71 | 3194.72 |
| 22 | 1870.682 | 1110 | 877.3772 | 737.68 | 666.03 | 485.92 | 583.72 | 591.61 |
| 23 | 11502.64 | 9694 | 9990.069 | 8944.62 | 8872.37 | 8468.94 | 7809.27 | 7985.69 |
| 24 | 5804.36 | 5629.6 | 5351.6 | 5066.27 | 6007.9 | 3667.71 | 4619.44 | 5168.32 |
| 25 | 5071 | 4707 | 4352.8 | 3917.8 | 4568.88 | 4256.83 | 4092.08 | 3193.41 |
| 26 | 1769.132 | 1010 | 1058.957 | 1112.5 | 949.92 | 1030.97 | 1310.02 | 1110.78 |
| 27 | 764.3513 | 935 | 714.181 | 575.44 | 517.37 | 469.49 | 436.77 | 523.13 |
| 28 | 2709.336 | 1804 | 1937.275 | 1472.54 | 1226.66 | 1337.26 | 1622.27 |  |
| 29 | 3594.614 | 3952 | 3764.117 | 3547.01 | 3225.55 | 3186.7 | 3107.19 | 2926.16 |
| 30 | 1446.74 | 1295.26 | 1164.98 | 1310.41 | 1207 | 1139 | 1258 | 1202 |
| 31 | 4995.03 | 4919.08 | 4478.04 | 4009.41 | 3770.14 | 4278.63 | 3352 | 3040.37 |
| 32 | 512.11 | 375.7 | 291.86 | 298.4 | 265.24 |  |  |  |
| 33 | 5486 | 5079 | 5437 | 5357 | 4422 | 4531.26 | 4807.25 | 4125 |
| 34 | 4702 | 4286.4 | 4249.8 | 3865 | 3410.73 | 3397.09 | 3443.26 | 3173.12 |
| 35 | 5750.4 | 4799.1 | 4331.2 | 4122.4 | 4406.19 | 3617.02 | 3705.68 | 3638.95 |
| 36 | 1741.4 | 1418.2 | 1347.4 | 1311 | 1289.21 | 1081.86 | 1146.77 | 1029.17 |
| 37 | 6389.3 | 4885.1 | 4735 | 4676.6 | 4539.69 | 4532.53 | 4114.86 | 3765.18 |
| 38 | 1215.5 | 1186.1 | 1116.7 | 781.65 | 766.8 | 934.6 | 848 | 1004 |
| 39 | 3289.7 | 3543.6 | 3763.4 | 3226.6 | 2778.91 |  |  |  |
| 40 | 923.1 | 562.3 | 584.4 | 479.3 | 430.43 | 452.94 | 493.6 | 393.49 |
| 41 | 3836.6 | 2150.9 | 1712 | 1592.9 | 1420.18 | 1089.03 | 1335.95 | 1147.67 |
| 42 | 2488.7 | 2177.6 | 2351.8 | 2702.9 | 1637.31 | 1871.56 | 1529.15 | 1464.02 |
| 43 | 1761.7 | 1578.8 | 1530.8 | 1505.99 | 1035.7 | 981.4 | 1204.7 | 1279 |
| 44 | 984.3 | 811.3 | 808.8 | 727.7 | 745.35 |  |  |  |
| 45 | 2903.3 | 3078.4 | 2607.9 | 2737.4 | 2688.9 |  |  |  |
| 46 | 6479.1 | 5366.8 | 5183.1 | 4376.7 | 3757.73 | 3509.99 | 3189.62 | 3348.66 |
| 47 | 5504.5 | 5406.6 | 4714.4 | 4517.4 | 3859.26 | 3300.26 | 3568.82 | 3463.01 |
| 48 |  |  | 5900 | 8659.2 | 7662.37 | 6679.15 | 6115.61 | 5220.69 |
| 49 | 434.4 | 294.8 | 370.5 | 263.3 | 351.01 |  |  |  |
| 50 | 3710 | 3310.3 | 2909.1 | 3063.1 | 2521.31 | 2301.37 | 2447.65 | 2150.55 |
| 51 | 1207 | 1553 | 1261 | 1032 | 959 | 826 | 1165 | 894 |
| 52 | 9279.043 | 8635 | 7913.188 | 7499.93 | 6467.76 | 6323.16 | 5984.02 | 5957.69 |
| 53 | 968 | 831.4 | 576.1 | 726.3 | 615.08 |  |  |  |
| 54 | 5221 | 4475.8 | 4149.9 | 3475.7 | 2670.17 | 2529.33 | 2691.37 | 2585.15 |
| 55 | 1658.89 | 1334 | 1495 | 1393.19 | 1151.53 |  |  |  |
| 56 | 1512.04 | 1105.16 | 963.3 | 952 | 864.6 |  |  |  |
| 57 | 4964.62 | 3748.97 | 3330.94 | 2972.92 | 2982.3 | 2381 | 2023.83 |  |
| 58 | 2164.36 | 1763.95 | 1488.41 | 1211.3 | 1153.5 |  |  |  |
| 59 | 2071.3 | 2046.8 | 1910.4 | 1869.67 | 1464.7 |  |  |  |
| 60 | 471.5 | 429.9 | 369 | 262.32 | 145.78 |  |  |  |
| 61 | 919 | 723 | 723 | 548 | 704 |  |  |  |
| 62 | 1585.9 | 1437.6 | 1324.3 | 1135.15 | 1154.7 | 1152.94 |  |  |
| 63 | 10523.3 | 9457.6 | 7743 | 6675.1 | 6153.22 | 5680.29 |  |  |
| 64 | 2576.45 | 2473.18 | 2033.79 | 2207.26 | 1306.84 |  |  |  |
| 65 | 1307.76 | 1166.87 | 1109.4 | 1120.27 | 1186.05 |  |  |  |
| 66 | 10130.2 | 9740.7 | 8876 | 7903.4 | 6858.83 | 6830.13 |  |  |
| 67 | 1594.225 | 1616 | 1200.268 | 897.72 | 471.35 | 581.76 | 805.52 | 660.52 |
| 68 | 1511.8 | 1429.7 | 1084.2 | 1014.5 | 1258.17 | 1193.77 | 1421.04 | 1278.51 |
| 69 | 5259.12 | 4075.34 | 4260.29 | 3845.04 | 3454.11 | 3286.49 | 2900.66 | 2958.99 |
| 70 | 1233.87 | 1252.74 | 1125.16 | 1154.48 | 1104.89 |  |  |  |
| 71 | 2064.826 | 2187 | 1988.614 | 1836.89 | 1526.49 | 1517.46 | 1232.77 | 1298.4 |
| 72 |  |  |  | 3112 | 2980.34 | 2518.73 | 3170.81 | 2816 |
| 73 | 843.5 | 926.4 | 721 | 623 | 688.89 | 456.37 | 501.24 | 501.57 |
| 74 | 5987.594 | 5518 | 5293.189 | 4873.3 | 4478.19 | 4353.25 | 4600.75 | 4165.03 |
| 75 | 15725.38 | 14963.86 | 15201 | 14275.2 | 15324.27 | 14180.8 | 13944.41 | 13407.68 |
| 76 | 7087.3 | 7141 | 5447.6 | 5645.7 | 6224.58 | 5707.63 | 5827.68 | 5425.6 |
| 77 | 14666.98 | 13352 | 11854.78 | 10244.56 | 9509.19 | 9142.84 | 8661.91 | 8630.79 |
| 78 | 3085 | 2754 | 2501 | 2140 | 2167 | 2213.5 | 1992 | 1174 |
| 79 | 2006.21 | 1990.2 | 980.91 | 947.74 | 778.08 | 725.22 | 572.91 | 793.08 |
| 80 | 11319.9 | 7813.7 | 7344.6 | 7463.4 | 6607.17 | 5141.67 | 4400.49 | 3986.42 |
| 81 | 5011 | 5817 | 5500 | 4342.5 | 4171.18 | 4026.2 | 4270.22 | 4337.07 |
| 82 | 12991.17 | 10251 | 8958.168 | 8034.8 | 7948.56 | 7800.13 |  |  |
| 83 | 3935.224 | 3227 | 2722.305 | 2315.79 | 2071.29 | 1860.4 | 1668.35 | 1384.84 |
| 84 | 2335.32 | 2174.3 | 2109.5 | 1886.86 | 1788.89 | 1369.98 | 1785.42 | 1073.28 |
| 85 | 2559.5 | 2497.1 | 2399.8 | 1779.9 | 2071.51 |  |  |  |
| 86 | 1684 | 1774.8 | 1040.4 | 1343.4 | 891.02 |  |  |  |
| 87 | 2187.6 | 2544 | 2442.1 | 2058.6 | 2054.8 | 1797.94 |  |  |
| 88 | 1731.62 | 1640.46 | 1488.64 | 1199.19 | 1005.17 | 1126.98 | 1384.65 | 1279.73 |
| 89 | 628.2 | 494.37 | 544.86 | 543.97 | 475.6 |  |  |  |
| 90 | 1893.92 | 1954 | 2072.483 | 1608.3 | 1697.33 | 1361.46 | 1439.71 | 1377.63 |
| 91 | 1147.6 | 1234.3 | 1004.1 | 947.4 | 908.75 |  |  |  |
| 92 | 5241.899 | 5070 | 4792.277 | 4565.99 | 4175.74 |  |  |  |
| 93 | 1198.88 | 1174.64 | 980.04 | 882.46 | 781.92 |  |  |  |
| 94 | 941.5 | 1000.5 | 898.4 | 766.5 | 691 | 654.92 | 607.28 | 610.85 |
| 95 | 3155.4 | 3202.3 | 2667.8 | 2885.3 | 2836.64 | 2819.32 | 2727.53 | 3288.57 |
| 96 | 10656.76 | 8956.09 | 9453.94 | 8298.31 | 8174.99 | 7654.08 | 7428.84 |  |
| 97 | 1238 | 1104 | 1058.3 | 1155.21 | 1316.64 | 1100.63 | 862.61 | 812.98 |
| 98 | 23496.7 | 21405.1 | 20357.9 | 18792.4 | 17705.18 | 16295.25 | 15137.3 | 14891.32 |
| 99 | 1865.667 | 1695 | 1607.5 | 1440.68 | 1378.69 |  |  |  |
| 100 | 3202.6 | 2559 | 2329.9 | 2015.8 | 1846.23 | 1939.1 | 1864.5 | 1878.21 |
| 101 | 3659.188 | 2528 | 2470.376 | 3093.23 | 2975.6 | 2678.5 |  |  |
| 102 | 11940 | 9150.84 | 8963.97 | 9047.78 | 8636.23 | 8255.01 | 7576.33 | 7023.24 |
| 103 | 858.7 | 654.6 | 601 | 536.4 | 489.41 |  |  |  |
| 104 | 22894.04 | 20120 | 18854.03 | 15873.32 | 15350.97 | 13814.36 | 12455.82 | 12145.47 |
| 105 | 10416.8 | 7665.7 | 6465.9 | 7015.4 |  |  | 4119.55 | 3760.38 |
| 106 | 3944 | 3232.11 | 3182.57 | 2680.49 | 1987.42 | 1512.58 | 1542.38 | 1089.05 |
| 107 | 3820.33 | 3150 | 2985.383 | 2741.06 | 2508.87 |  |  |  |
| 108 | 1998.8 | 2018.8 | 1830.8 | 1989.2 | 1924.04 |  |  |  |
| 109 | 3628 | 3479.1 | 3662.2 | 3291.8 | 2932.12 |  |  |  |
| 110 | 9042 | 8064 | 7433 | 7778 | 7064.77 | 5428.14 | 4528.34 | 4862.25 |
| 111 | 997.12 | 1143.14 | 1004.3 | 858.98 | 853.54 | 663 | 713.38 | 653.28 |
| 112 | 5534.7 | 3189.79 | 2803.22 | 2371.92 | 2479.59 | 2487.36 | 2469.45 | 2141.39 |
| 113 | 22687.37 | 22494.82 | 20616.02 | 19254.46 | 18451.35 | 17764.36 | 11812.15 | 16989.7 |
| 114 | 660 | 806.94 | 737.76 | 563.23 | 548 | 429 | 426.9 |  |

Note: The empty cell means that the annual methane collection rate was not reported/available.
